# Supplementary material for: Chemo-enzymatic cascades to produce cycloalkenes from bio-based resources
Source: Nat Commun. 2019 Nov 7;10:5060. doi: 10.1038/s41467-019-13071-y (PMC6838201; doi:10.1038/s41467-019-13071-y)
Supplement: Supplementary file 1 — Supplementary Information [file 41467_2019_13071_MOESM1_ESM.pdf]

Supplementary Information for:

Chemo-enzymatic cascades to produce cycloalkenes from bio-based resources

Shuke Wu[1,3]*, Yi Zhou[1], Daniel Gerngross[2], Markus Jeschek[2] and Thomas R. Ward[1]*

[1] Department of Chemistry, University of Basel, Mattenstrasse 24a, BPR 1096, CH-4058 Basel, Switzerland.

[2] Department of Biosystems Science and Engineering, ETH Zurich, Mattenstrasse 26, CH-4058 Basel, Switzerland.

[3] Current address: Institute of Biochemistry, University of Greifswald, Felix-Hausdorff-Str. 4, D-17489 Greifswald, Germany.

*e-mail: thomas.ward@unibas.ch, shukewu@u.nus.edu

Table of Contents

Supplementary Figures 3

Supplementary Table 24

Supplementary Methods 25

Chemicals and Materials 25

Analytical Methods 26

DNA Sequences 26

Genetic Engineering of *E. coli* Expressing a Single Enzyme 31

Genetic Engineering of Different Tagged PfBVMOs and PpBVMOs 32

Engineering of E. coli with Combinatorial RBS Libraries 35

Screening of *E. coli* Libraries for the production of 4b and 4a 36

Procedure for Decarboxylation of 4a-4c and 3a-3c 37

Procedure for Metathesis of 2a-2c 37

Procedure for Chemoenzymatic Conversion of 4a-4c to 1a-1c 38

Procedure for Chemoenzymatic Conversion of 6 to 1a-1c 38

Procedure for Chemoenzymatic Conversion of 7 to 1a-1c 39

Supplementary References 39

Supplementary Figures

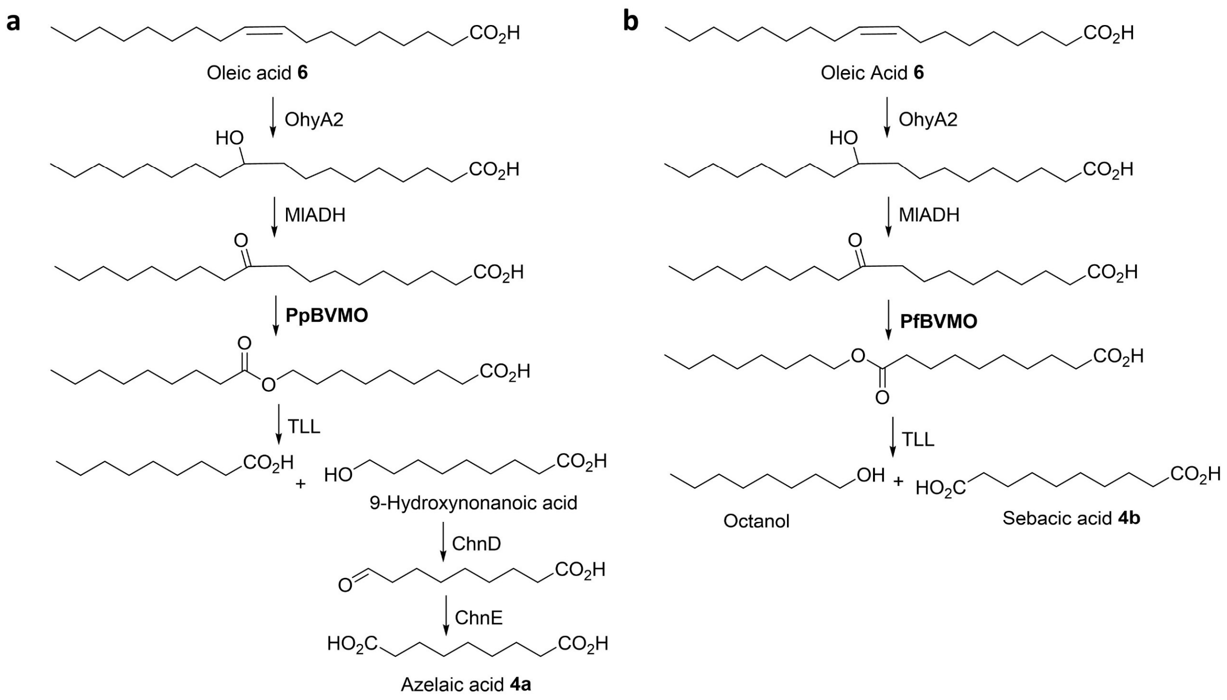

Supplementary Figure 1. Enzyme cascades for the conversion of oleic acid (6) to diacids (4a,4b). **a**, Conversion of oleic acid (**6**) to azelaic acid (**4a**) via hydration by OhyA2, oxidation by MlADH, Baeyer–Villiger oxidation by PpBVMO, hydrolysis by TLL and further oxidation by ChnD and ChnE[1,2]. **b**, Conversion of oleic acid (**6**) to sebacic acid (**4b**) via hydration by OhyA2, oxidation by MlADH Baeyer–Villiger oxidation by PfBVMO and hydrolysis by TLL[1].

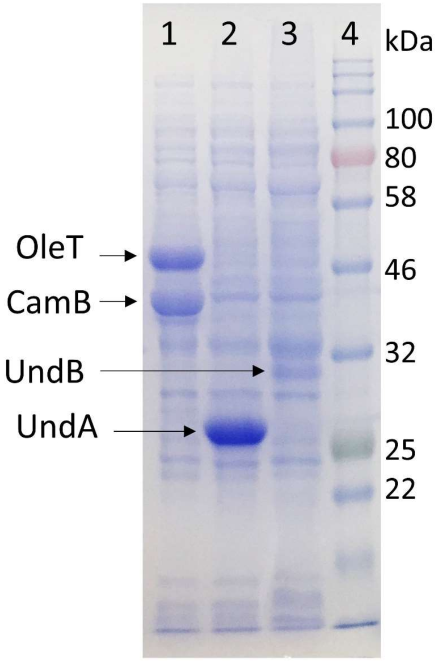

Supplementary Figure 2. SDS-PAGE analysis of *E. coli* expressing different decarboxylases. Lane 1: *E. coli* (OleT-CamAB); Lane 2: *E. coli* (UndA); Lane 3: *E. coli* (UndB); Lane 4: protein molecular weight standard. Source data are provided as a Source Data file.

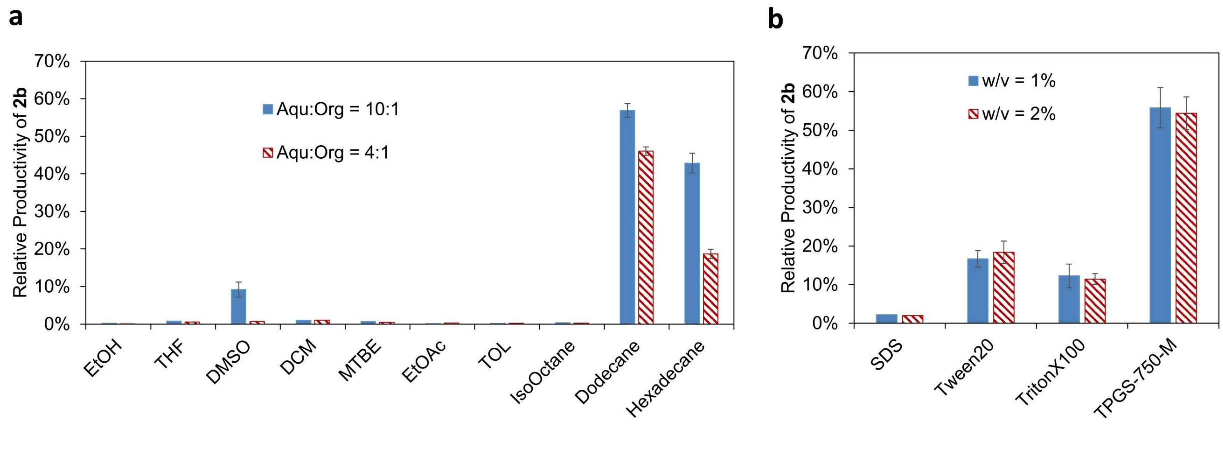

Supplementary Figure 3. Decarboxylation of 4b with *E. coli* (UndB) in different reaction systems. a, Reactions were performed in KP buffer (200 mM, pH 8.0) with different organic solvents (v:v = 10:1 or 4:1). **b**, Reactions were performed in KP buffer (200 mM, pH 8.0) with different surfactants (w:v = 1% or 2%). Reaction conditions: **4b** (5 mM), *E. coli* (UndB) (10 g l^{-1}) in KP buffer (0.5 ml, 200 mM pH 8.0, 1% glucose) with organic solvent (50-125 µl) or surfactant (1-2%), 30 °C, 250 rpm, 24 h. Source data are provided as a Source Data file. Data are mean values of duplicate experiments with error bars indicating standard deviations (n = 2).

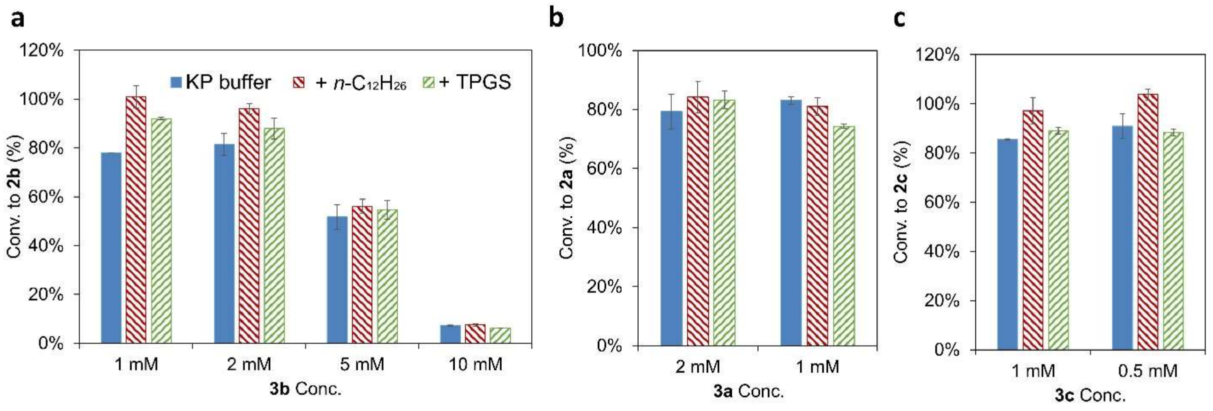

Supplementary Figure 4. Bis-decarboxylation of diacids (4a-4c) at 0.5-10 mM with *E. coli* (UndB). a, Conversion of **4b** (1-10 mM) to **2b**. **b**, Conversion of **4a** (1-2 mM) to **2a**. **c**, Conversion of **4c** (0.5-1 mM) to **2c**. Reaction conditions: *E. coli* (UndB) cells (10 g l^{-1}), KP buffer (200 mM pH 8.0, 1% glucose) with or without *n*-dodecane (10%) or TPGS-750-M (1%), 30 °C, 250 rpm, 24 h. Source data are provided as a Source Data file. Data are mean values of duplicate experiments with error bars indicating standard deviations (n = 2).

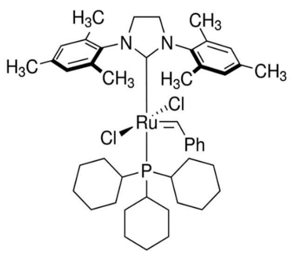

Ru1

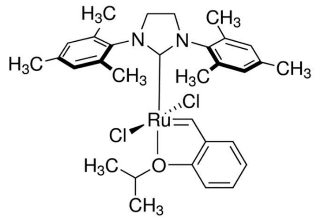

Ru2

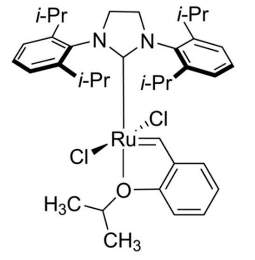

Ru3

Supplementary Figure 5. Structures of the ruthenium catalysts investigated in this study.

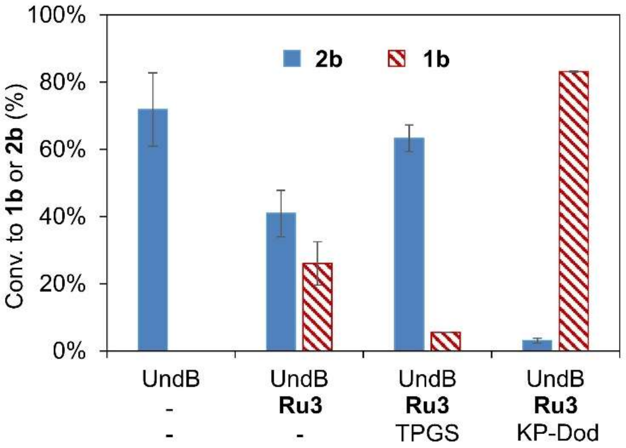

Supplementary Figure 6. Sequential cascade for converting sebacic acid (4b) to cyclohexene (1b). Reaction conditions: **4b** (2 mM), *E. coli* (UndB) cells (10 g l^{-1}), **Ru3** (100 μM), KP buffer (200 mM pH 8.0, 1% glucose) with or without *n*-dodecane (10%) or TPGS-750-M (1%), 30 °C, 250 rpm, 24 h. Source data are provided as a Source Data file. Data are mean values of duplicate experiments with error bars indicating standard deviations (n = 2).

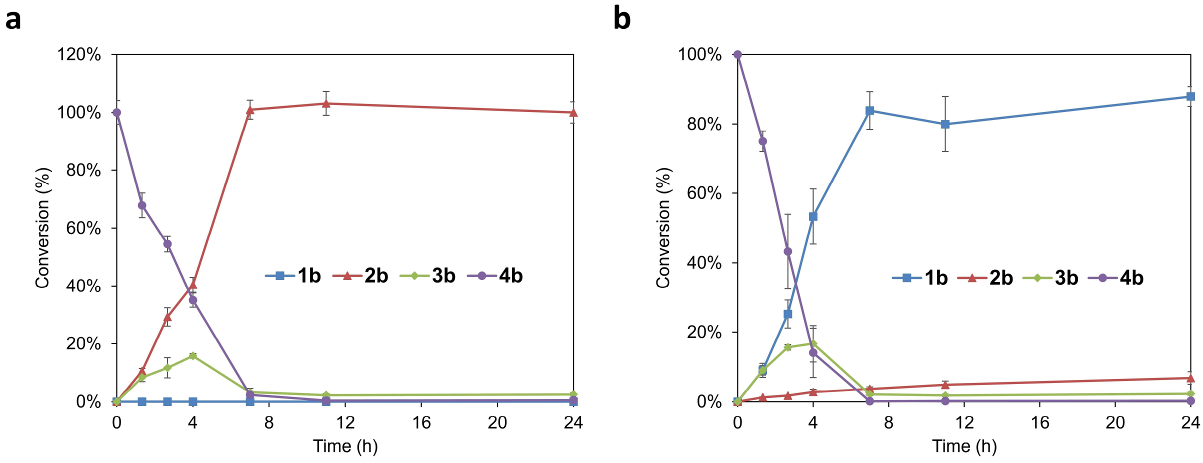

Supplementary Figure 7. Reaction progress of decarboxylation and decarboxylation-metathesis. a, bis-decarboxylation of diacid (**4b**) to diene (**2b**) using *E. coli* (UndB) cells. **b**, Concurrent bis-decarboxylation-metathesis for the conversion of diacid (**4b**) to cyclohexene (**1b**) with *E. coli* (UndB) cells and **Ru3**. Reaction conditions: **4b** (2 mM), *E. coli* (UndB) cells (10 g l^{-1}), **Ru3** (for b, 100 μM), KP buffer (200 mM pH 8.0, 1% glucose) with *n*-dodecane (10%), 30 °C, 250 rpm, 24 h. The progress of the reaction was monitored by GC-MS using acetophenone as internal standard (see Supplementary Figure 16 for calibration curve and Supplementary Figure 18 for representative GC traces). Source data are provided as a Source Data file. Data are mean values of triplicate experiments with error bars indicating standard deviations (n = 3).

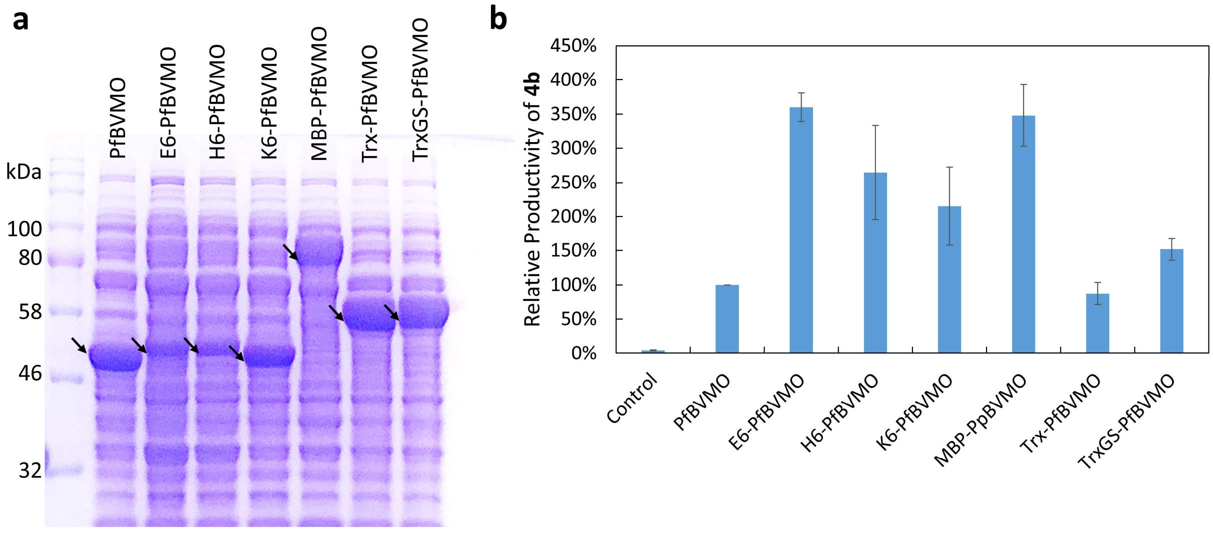

Supplementary Figure 8. PfBVMOs with different N-terminal tags for converting oleic acid (6). a, SDS-PAGE analysis of whole-cell protein extracts of *E. coli* expressing PfBVMO with different N-terminal tags. E6: 6x Glu tag; H6: 6x His tag; K6: 6x Lys tag; MBP: maltose-binding protein from *E. coli*; Trx: Thioredoxin from *E. coli*; TrxGS: Thioredoxin with a GGSGGGGSGG linker. **b**, Production of sebacic acid (**4b**) from oleic acid (**6**) by using a mixture of *E. coli* cells expressing OhyA2, MlADH, TLL, and different PfBVMOs. Reaction conditions: **6** (5 mM), *E. coli* (OhyA2) (5 g l^{-1}), *E. coli* (MlADH) (5 g l^{-1}), *E. coli* (TLL) (5 g l^{-1}), *E. coli* expressing different PfBVMOs (10 g l^{-1}), KP buffer (200 mM pH 8.0, 1% glucose), 30 °C, 250 rpm, 24 h. Control is the reaction with *E. coli* (OhyA2), *E. coli* (MlADH) and *E. coli* (TLL), but without *E. coli* expressing PfBVMO. Source data are provided as a Source Data file. Data in **b** are mean values of triplicate experiments with error bars indicating standard deviations (n = 3).

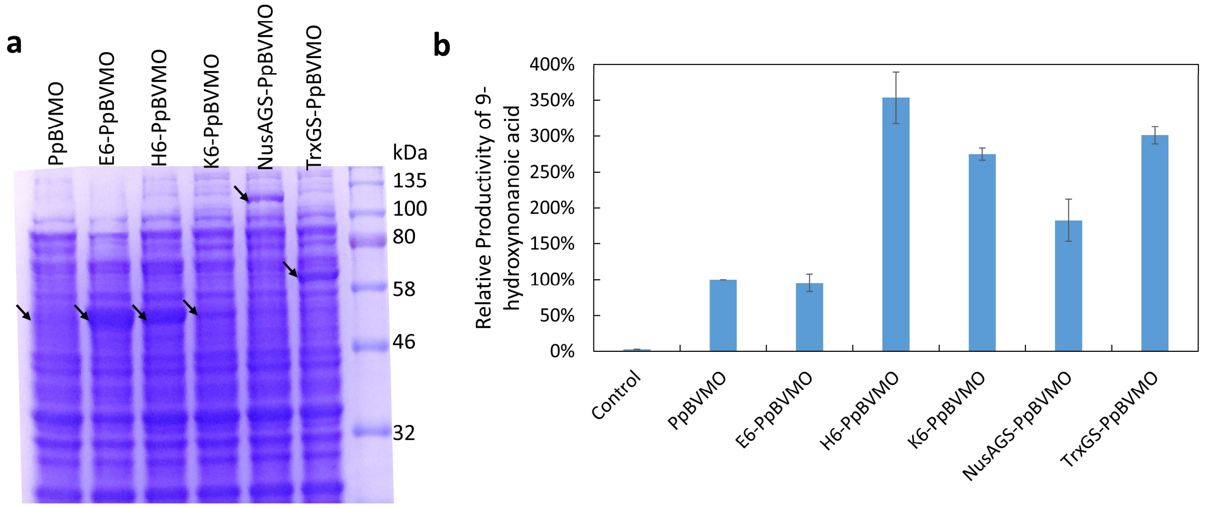

Supplementary Figure 9. PpBVMOs with different N-terminal tags for converting oleic acid (6). a, SDS-PAGE analysis of whole-cell protein extracts of *E. coli* expressing PpBVMO with different N-terminal tags. E6: 6x Glu tag; H6: 6x His tag; K6: 6x Lys tag; NusA: N-utilization substance protein A from *E. coli*; TrxGS: Thioredoxin with a GGSGGGGSGG linker. **b**, Production of 9-hydroxynonanoic acid from oleic acid (**6**) by using a mixture of *E. coli* cells expressing OhyA2, MlADH, TLL, and different PfBVMOs. Reaction conditions: **6** (5 mM), *E. coli* (OhyA2) (5 g l^{-1}), *E. coli* (MlADH) (5 g l^{-1}), *E. coli* (TLL) (5 g l^{-1}), *E. coli* expressing different PpBVMOs (10 g l^{-1}), KP buffer (200 mM pH 8.0, 1% glucose), 30 °C, 250 rpm, 24 h. Control is the reaction with *E. coli* (OhyA2), *E. coli* (MlADH) and *E. coli* (TLL), but without *E. coli* expressing PpBVMO. Source data are provided as a Source Data file. Data in **b** are mean values of triplicate experiments with error bars indicating standard deviations (n = 3).

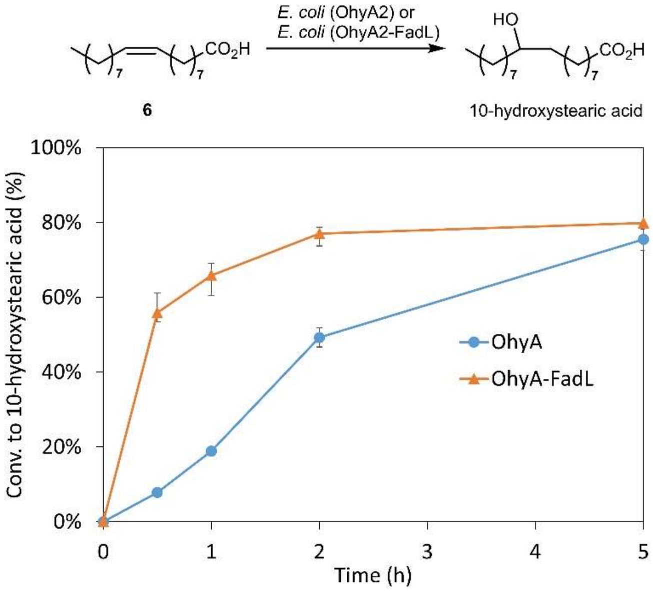

Supplementary Figure 10. Reaction progress of hydration of oleic acid (6) to 10-hydroxystearic acid. Reaction conditions: **6** (20 mM), *E. coli* (OhyA2) or *E. coli* (OhyA2-FadL) (5 g l^{-1}), KP buffer (200 mM pH 8.0, 1% glucose), 30 °C, 250 rpm, 5 h. The progress of the reaction was monitored by GC-MS and the conversions were estimated using the relative peak areas on the total ion chromatogram. Source data are provided as a Source Data file. Data are mean values of triplicate experiments with error bars indicating standard deviations (n = 3).

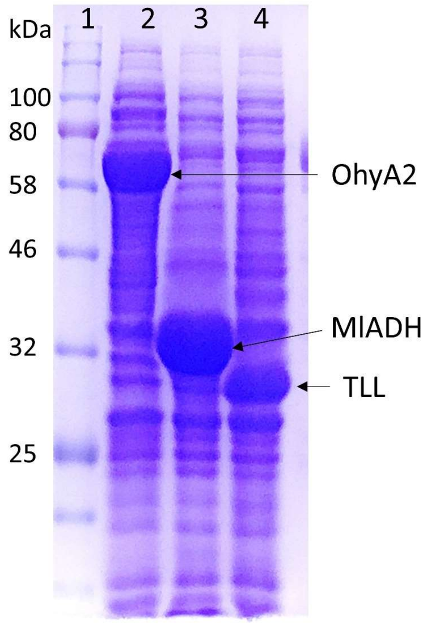

Supplementary Figure 11. SDS-PAGE analysis of *E. coli* expressing different enzymes. Lane 1: protein marker; Lane 2: *E. coli* (OhyA2); Lane 3: *E. coli* (MlADH); Lane 4: *E. coli* (TLL). Source data are provided as a Source Data file.

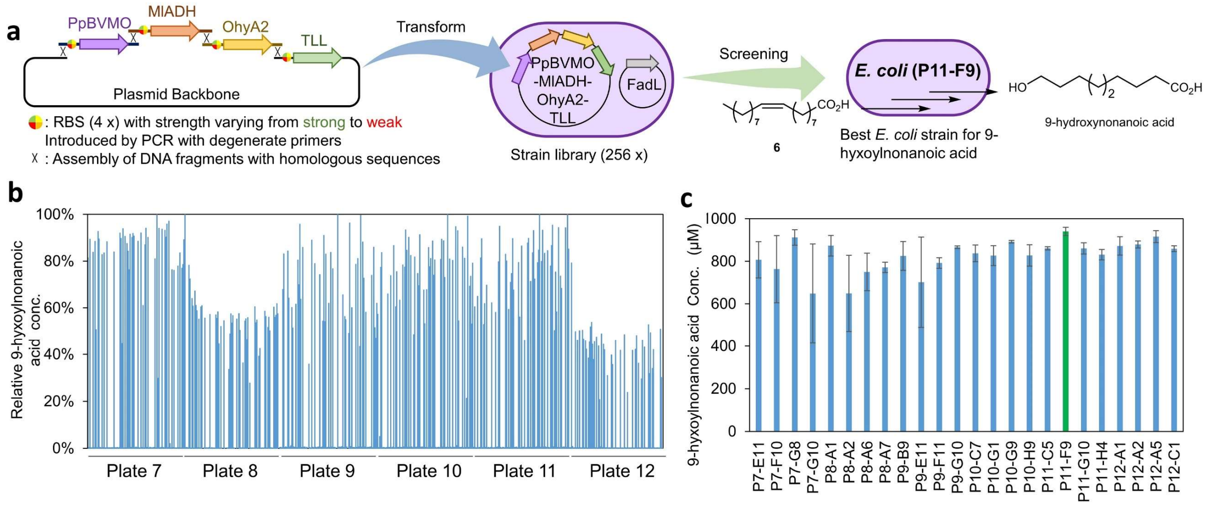

Supplementary Figure 12. A strain library for the production of 9-hydroxynonanoic acid from 6. a, Construction of a plasmid library for the co-expression of PpBVMO, MlADH, OhyA2, and TLL with different expression levels, and screening of the resulting *E. coli* library to identify the most effective strain for the production of 9-hydroxynonanoic acid. All *E. coli* strains included an additional plasmid harboring the fatty acid transporter FadL. **b,** Initial screening of 576 strains in six 96-well plates for production of 9-hydroxynonanoic acid. **c**, Further validation and comparison of 24 strains (best four from each plate) for the production of 9-hydroxynonanoic acid. The reactions were performed using oleic acid (**6**, 5 mM) and *E. coil* (10 g l^{-1}) in KP buffer (200 mM, pH 8.0, 1% glucose) at 30°C for 24 h. Data in **c** are mean values of triplicate experiments with error bars indicating standard deviations (n = 3). The quantification of 9-hydroxynonanoic acid was performed by UPLC-MS, using benzyl alcohol as internal standard. See Supplementary Figure 21 for the calibration curve. Source data are provided as a Source Data file. Data in **c** are mean values of triplicate experiments with error bars indicating standard deviations (n = 3).

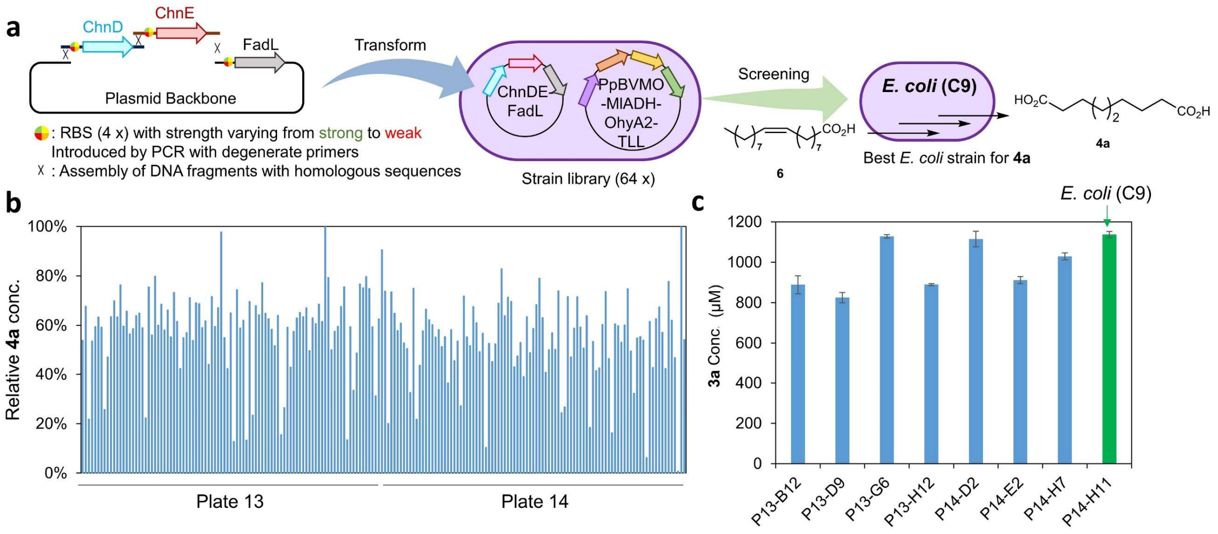

Supplementary Figure 13. A strain library for the production of azelaic acid (4a) from oleic acid (6). **a,** Construction of a plasmid library for the co-expression of ChnD, ChnE and FadL with different expression levels, and screening of the resulting *E. coli* library to identify the most effective strain for the production of azelaic acid (**4a**). All *E. coli* strains included an additional plasmid PpBVMO-MlADH-OhyA2-TLL from the strain P11-F9 in Figure S12. **b,** Initial screening of 192 strains in two 96-well plates for production of azelaic acid (**4a**). **c**, Further validation and comparison of 8 strains (best four from each plate) for the production of azelaic acid (**4a**). The reactions were performed using oleic acid (**6**, 5 mM) and *E. coil* (10 g l^{-1}) in KP buffer (200 mM, pH 8.0, 1% glucose) at 30°C for 24 h. Data in **c** are mean values of triplicate experiments with error bars indicating standard deviations (n = 3). The quantification of azelaic acid was performed by UPLC-MS, using benzyl alcohol as internal standard. See Supplementary Figure 21 for the calibration curve. Source data are provided as a Source Data file. Data in **c** are mean values of triplicate experiments with error bars indicating standard deviations (n = 3).

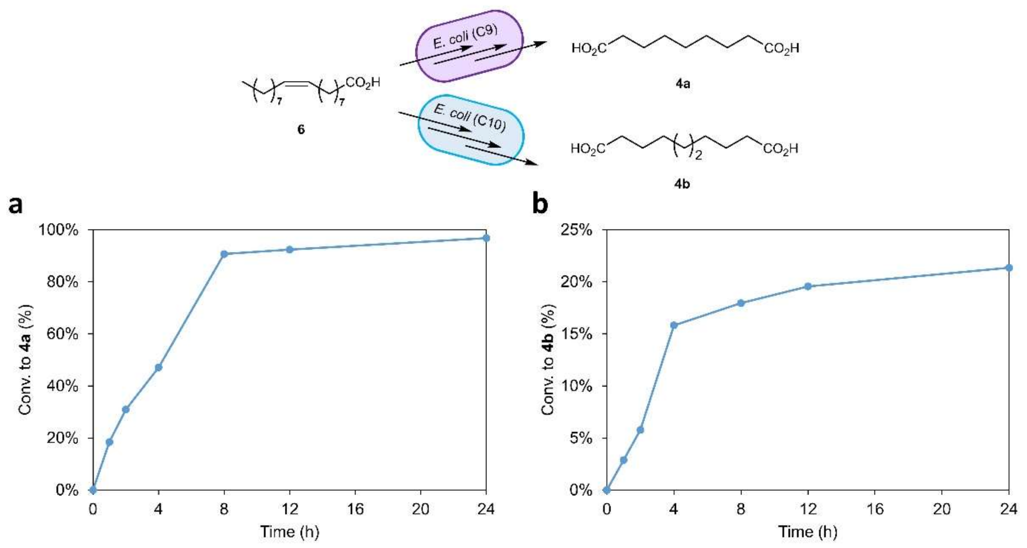

Supplementary Figure 14. Reaction progress of enzyme cascades for converting 6 to 4a and 4b. a, Conversion of oleic acid (**6**) to azelaic acid (**4a**) using *E. coli* (C9) cells. **b**, Conversion of oleic acid (**6**) to sebacic acid (**4b**) using *E. coli* (C10) cells. Reaction conditions: **6** (2 mM), *E. coli* whole cells (10 g l^{-1}), KP buffer (200 mM pH 8.0, 1% glucose), 30 °C, 250 rpm, 24 h. The quantification of azelaic acid and sebacic acid was performed by GC-MS using acetophenone as internal standard (see Supplementary Figure 16 for the calibration curves). Source data are provided as a Source Data file.

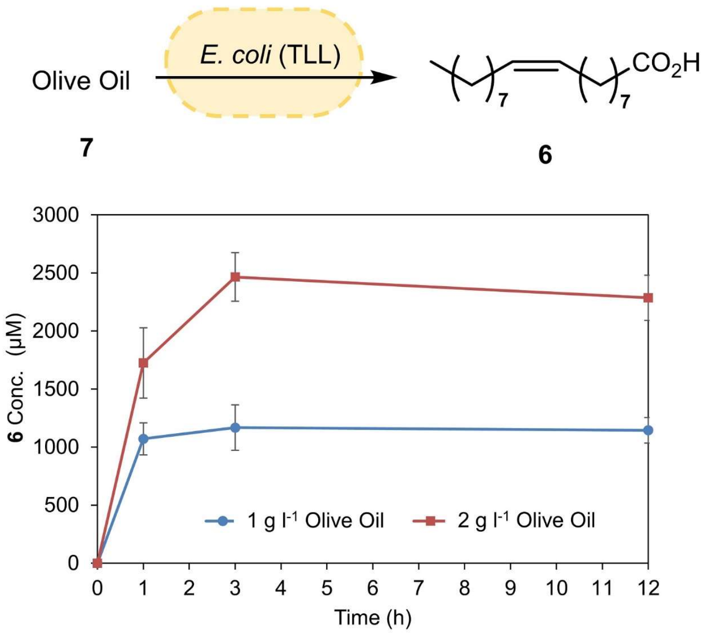

Supplementary Figure 15. Reaction progress of hydrolysis of 7 to 6 using *E. coli* (TLL) cells. Reaction conditions: **7** (1-2 g l^{-1}), lyophilized *E. coli* (TLL) cells (2 g l^{-1}), KP buffer (200 mM pH 8.0), 30 °C, 250 rpm, 12 h. The quantification of oleic acid was performed by UPLC-MS, using benzyl alcohol as the internal standard. See Supplementary Figure 21 for the calibration curve. Source data are provided as a Source Data file. Data are mean values of triplicate experiments with error bars indicating standard deviations (n = 3).

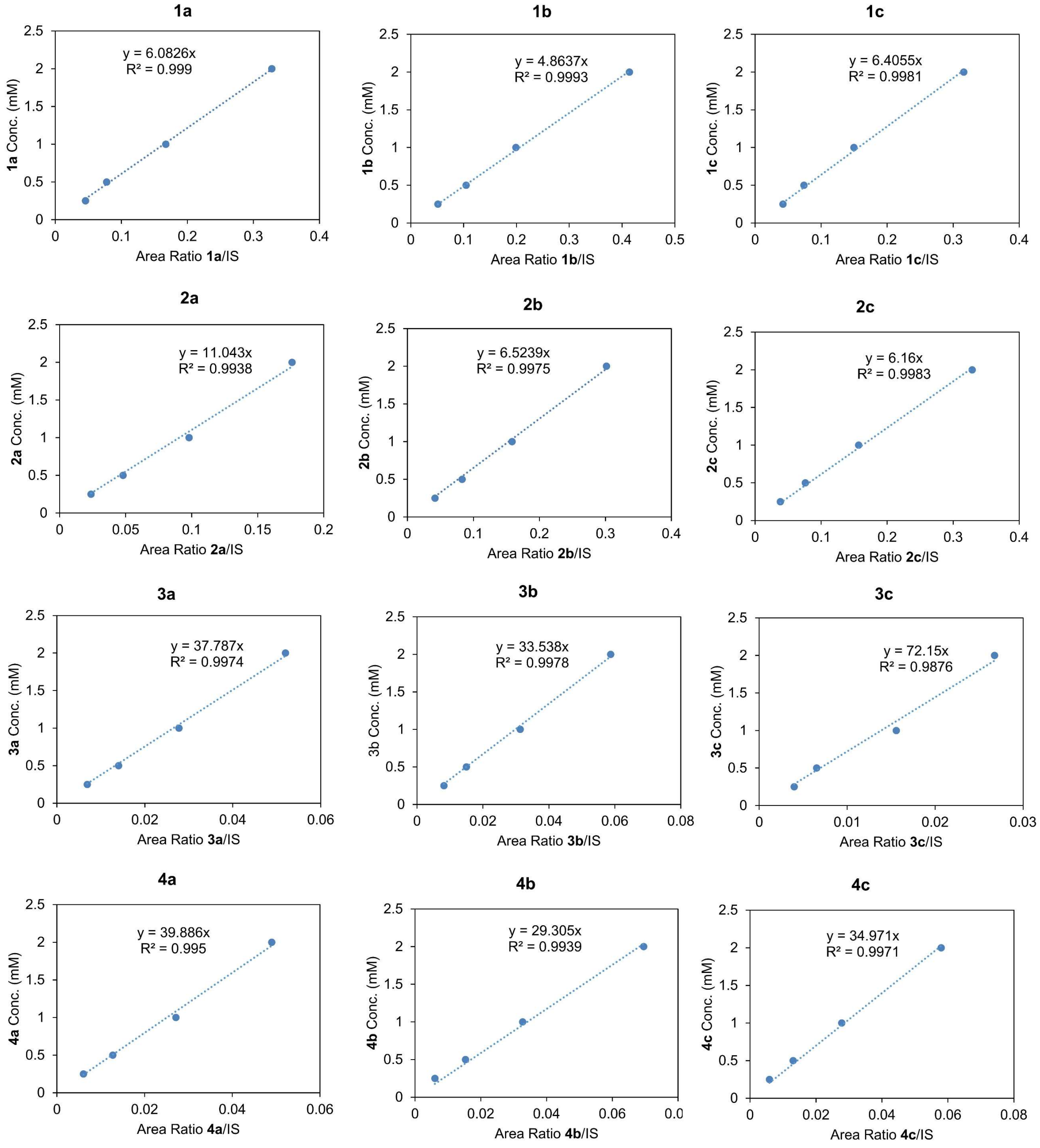

Supplementary Figure 16. Calibration curves of 1a-1c, 2a-2c, 3a-3c, and 4a-4c by GC-MS. (See Supplementary Methods for detailed GC conditions) Source data are provided as a Source Data file.

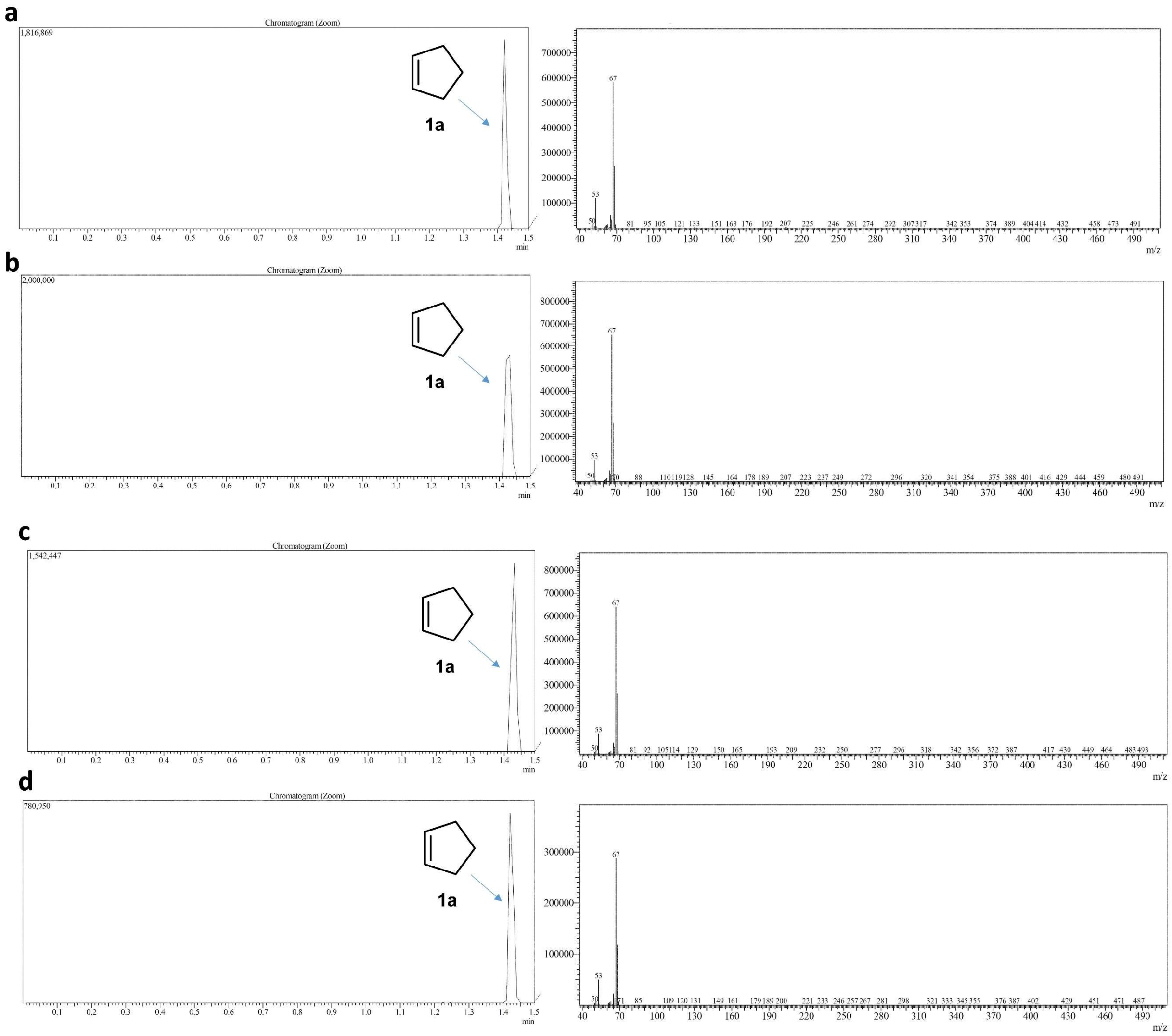

Supplementary Figure 17. Representative GC-MS chromatograms and MS spectra of 1a. The chromatograms are under selective ion monitoring (m/z = 67) and MS spectra are the target peak of **1a** at 1.43 min. **a**, **1a** standard. **b**, **1a** produced from **4a**. **c**, **1a** produced from **6**. **d**, **1a** produced from **7**.

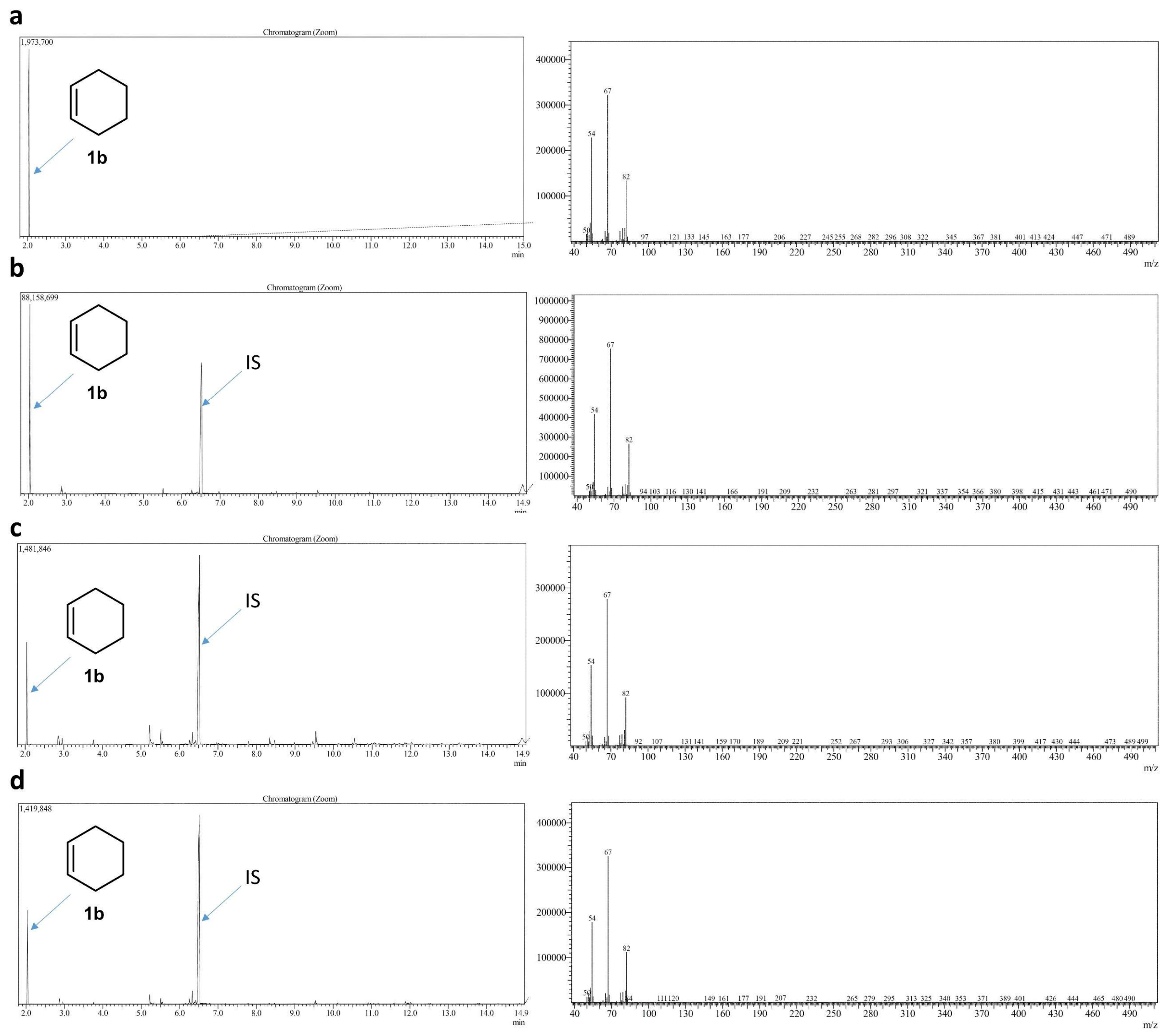

Supplementary Figure 18. Representative GC-MS chromatograms and MS spectra of 1b. The chromatograms are under selective ion monitoring (m/z = 67) and MS spectra are the target peak of **1b** at 2.04 min. **a**, **1b** standard. **b**, **1b** produced from **4b**. **c**, **1b** produced from **6**. **d**, **1b** produced from **7**.

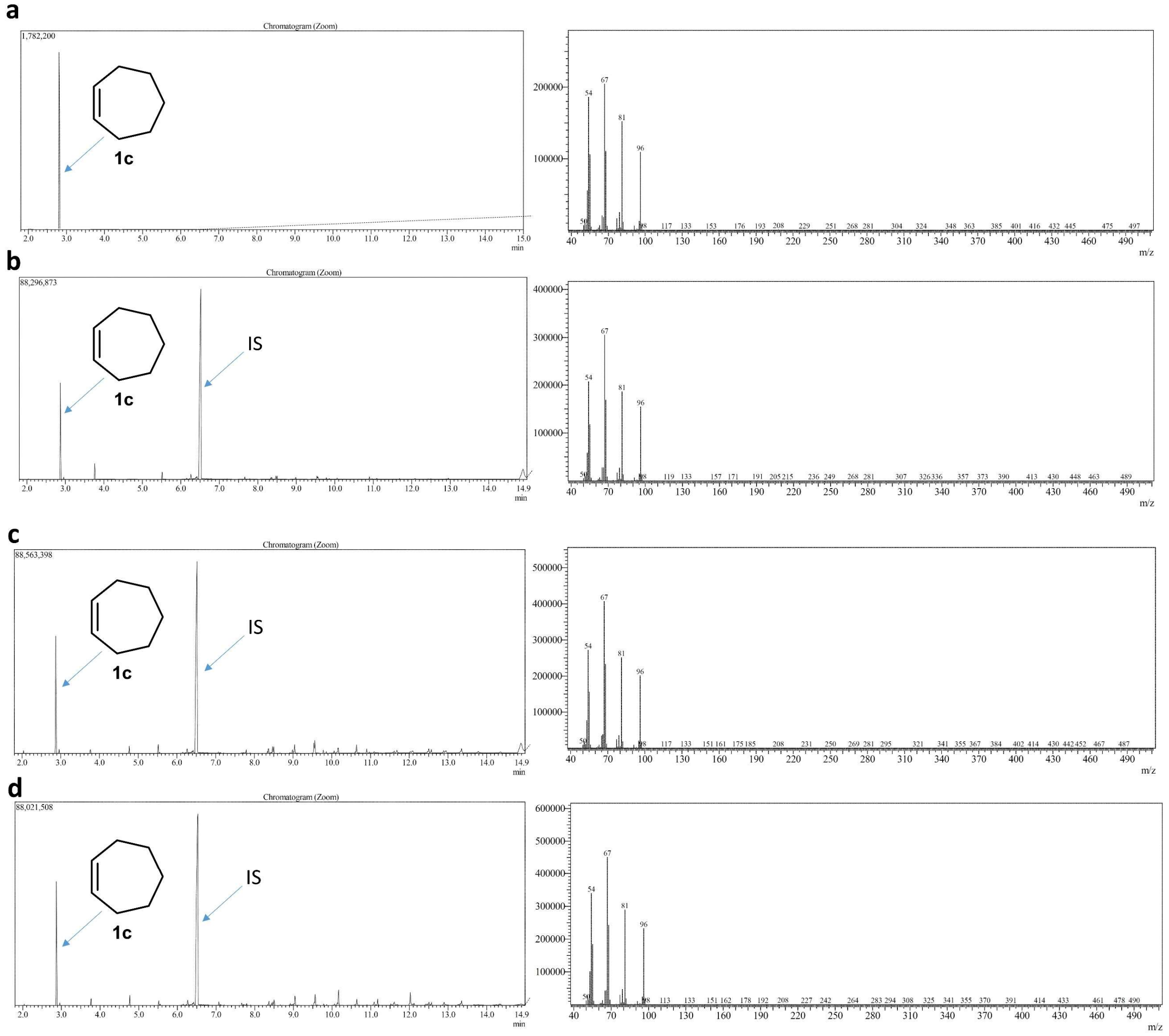

Supplementary Figure 19. Representative GC-MS chromatograms and MS spectra of 1c. The chromatograms are under selective ion monitoring (m/z = 67) and MS spectra are the target peak of **1c** at 2.88 min. **a**, **1c** standard. **b**, **1c** produced from **4c**. **c**, **1c** produced from **6**. **d**, **1c** produced from **7**.

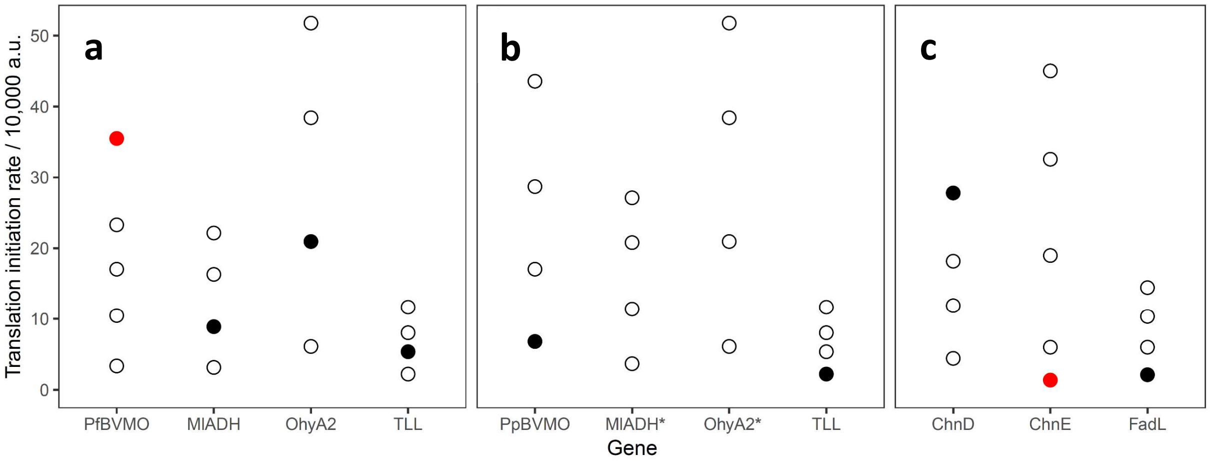

Supplementary Figure 20. Rationally reduced RBS libraries designed in silico by RedLibs. Predicted translation initiation rates (TIRs) of the designed RBSs are depicted for **a**) the PfBVMO-MlADH-OhyA2-TLL operon, **b**) the PpBVMO-MlADH-OhyA2-TLL operon, and **c**) the ChnD-ChnE-FadL operon, respectively. Library members that were found in the best performing strains after screening are highlighted by closed circles. The best performing RBSs for PfBVMO and ChnE were found to contain a 10 bp insertion and a point mutation, respectively, which affects the corresponding predicted TIRs significantly. These mutants were therefore included with the corrected TIRs and highlighted in red. (*) Mutant P11-F9 (panel **b**) was found to contain a mutation in the stop codon of MlADH which results in an MlADH-OhyA2 fusion protein. As a consequence, the actual relative translation rates for the corresponding genes could not be reliably predicted for this clone. For a detailed description of in silico RBS library design please refer to the Supplementary Methods. Source data are provided as a Source Data file.

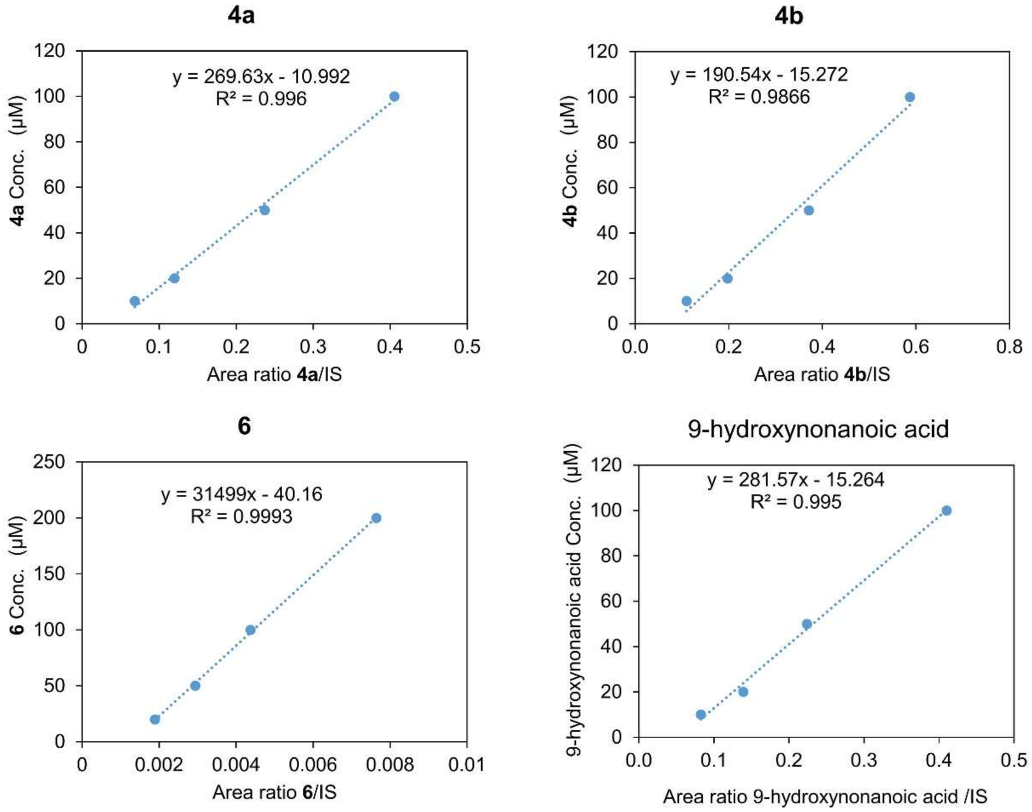

Supplementary Figure 21. Calibration curves of 4a, 4b, 6 and 9-hydroxynonanoic acid (UPLC-MS). (See Supplementary Methods for detailed UPLC-MS conditions) Source data are provided as a Source Data file.

Supplementary Table

Supplementary Table 1. List of primers used in this study.

**Name**	**Sequence**
UndA-NcoI-F	ACTGCCATGGAAATCACCCGTATTAAAG
UndB-BspHI-F	ACTGTCATGAGCCCGAGCCCGGCTTCAC
pET28a-XhoI-R	TGGTGGTGGTGGTGCTCGAG
MBP-BspHI-F	ACTGTCATGAAAATCGAAGAAGGTAAACTGGTAATC
MBP-NdeI-R	ACTGCATATGCTTGGTGATACGAGTCTGCGCGTCT
NusA-BspHI-F	ACTGTCATGAACAAAGAAATTTTGGCTGTAGTTG
NusA-NdeI-R	ACTGCATATGCGCTTCGTCACCGAACCAGCAAATA
Trx-BspHI-F	ACTGTCATGAGCGATAAAATTATTCACCTGAC
Trx-NdeI-R	ACTGCATATGCGCCAGGTTAGCGTCGAGGAACTCT
PfBVMO-BspHI-F	ACTGTCATGAACGCCCATAGCGATAGCATTG
PfBVMO-NcoI-E6-F	ACTGCCATGGAAGAAGAAGAAGAAGAAATGAACGCCCATAGCGATAGCAT
PfBVMO-BspHI-K6-F	ACTGTCATGAAGAAAAAAAAGAAAAAGATGAACGCCCATAGCGATAGCAT
PfBVMO-NdeI-GS-F	ATCATATGGGTGGTTCTGGCGGTGGCGGTTCTGGTGGTAACGCCCATAGCGATA GCATTG
PpBVMO-BspHI-F	ACTGTCATGAGCAGTCATACCGCACTGC
PpBVMO-NcoI-E6-F	ACTGCCATGGAAGAAGAAGAAGAAGAA ATGAGCAGTCATACCGCACTGC
PpBVMO-BspHI-K6-F	ACTGTCATGAAGAAAAAAAAGAAAAAG ATGAGCAGTCATACCGCACTGC
PpBVMO-NdeI-GS-F	ACTGCATATGGGTGGTTCTGGCGGTGGCGGTTCTGGTGGTAGCAGTCATAC CGCACTGC
TLL-BspHI-F	ACTGTCATGAGTCCTATTCGTCGAGAGG
TLL-XhoI-R	ACTGCTCGAGTTAAAGACATGTCCCAATTAACCCG
FadL-BspHI-F	ACTGTCATGAGCCAGAAAACCCTGTTTACAAAG
FadL-XhoI-R	ACTGCTCGAGTCAGAACGCGTAGTTAAAGTTAGTAC
RBS4-PfBVMO-F	ATTTTGATAAGCCACGTTAAGGAGG**W**TGT**K**G ATGGAAGAAGAAGAAGAAGAAATGA
PfBVMO-RBS4-R	TCCAAGTCCCATTATTTAACTCAGGCTACCTTCGG
RBS4-MlADH-F	CCTGAGTTAAATAATGGGACTTGGA**S**CC**M**AAAAGGAGGTTAAC ATGAGCGAATTTACCCGTTTTGAAC
MlADH-RBS4-R	GTACCTCCTTATTATTATTATAGCTTAACCCAGCGGGGTATCTTTATC
RBS4-OhyA-F	A GCTATAATAATAATAAGGAGGTAC**WK**ATGAGTCAGCCGACCGCCCCGG
OhyA-RBS4-R	CACCTCCTTTACTAATACTGCCCTTAAGGAGCACGACGGCGACCCAGG
RBS4-TLL-F	AA GGGCAGTATTAGTAAAGGAGGTG**YM**AATGAGTCCTATTCGTCGAGAGG
pRSF-RBS4-R	CCTCCTTAACGTGGCTTATCAAAATTATTTCTACAGGGGAATTGT
PpBVMO-RBS4-F	GAAACCAAACGTACTTAAGGAGGTCA**MK**TATGGGCAGCAGCCATCATCATCATC
PpBVMO-RBS4-R	CCTACTATCTGGCCGTCCTGCTATCTTAACGACGGCTACCCTGCTGTTG
RBS4-MlADH-F2	GATAGCAGGACGGCCAGATAGTAGGGAGGT**N**ATTGATGAGCGAATTTACCCGTTTTGAAC
pRSF-RBS4-R2	GACCTCCTTAAGTACGTTTGGTTTCTCTAGAGGGGAATTGTTATCCGCTCACAAT
ChnD-RBS4-F	TAGATGAGTTTATTCTTTAAGGAGG**Y**G**W**TTAAATGCATTGCTATTGCGTTACCCAT
RBS4-ChnD-R	ATTTAGTTTAATTTTCATGCATCAGAACAATGCG
ChnE-RBS4-F	CTGATGCATGAAAATTAAACTAAAT**WW**AGGAGGTCCTAAATGAACTACCCGAATATTCCGCTG
RBS4-ChnE-R	TTATTTATGATCGCCGAACTTAATATTAATTCAGCTGGGTAATGAATTTGGTG
FadL-RBS4-F	TATTAAGTTCGGCGATCATAAATAAGGAGG**YK**TGTTCATGAGCCAGAAAACCCTGTTTACAAAG
pACYC-RBS4-R	CCTCCTTAAAGAATAAACTCA TCTAGAGGGGAATTGTTATCCGCTCACAAT

Supplementary Methods

Chemicals and Materials

All the chemicals were purchased from commercial suppliers and used without further purification. The key chemicals are listed below.

Chemicals from Sigma-Aldrich: cyclopentene **1a** (96%), cyclohexene **1b** (99%), cycloheptene **1c** (97%), 1,6-heptadiene **2a** (99%), 1,8-nonadiene **2c** (99%), 7-octenoic acid **3a** (97%), 8-nonenoic acid **3b** (97%), 9-decenoic acid **3c** (95%), sebacic acid **4b** (99%), undecanedioic acid **4c** (97%), oleic acid **6** (98%), Grubbs catalyst 2nd generation **Ru1**, Hoveyda-Grubbs catalys 2nd generation **Ru2**, Grubbs catalyst C711 **Ru3**, *n*-dodecane (99%), TPGS-750-M (DL-α-tocopherol methoxypolyethylene glycol succinate), acetophenone (99%), (trimethylsilyl)diazomethane solution 2.0 M in diethyl ether, glucose (99%).

Chemicals from Acros Organics: 1,7-octadiene **2b** (99%), azelaic acid **4a** (98%), olive oil **7** (pure, refined), KH_2PO_4, K_2HPO_4, Na_2HPO_4, NaH_2PO_4.

Chemicals from Fisher Scientific: MeOH (LC-MS grade), ACN (LC-MS grade), EtOAc (GC-MS grade). formic acid (LC-MS grade).

Fast digest restriction enzymes and T4 DNA ligase were purchased from Thermo Fisher.

Q5 high fidelity DNA polymerase and NEBuilder HiFi DNA assembly master mix were purchased from New England Biolabs

Plasmid miniprep-kit and gel extraction-kit were bought from Macherey-Nagel.

DNA primers were ordered from Microsynth.

LB (Lysogeny broth) medium was used for genetic engineering of *E. coli*.

A modified M9 medium supplemented with glucose (20 g l^{-1}), and yeast extract (6 g l^{-1}) was used for culturing *E. coli* cells for whole-cell reactions. The M9 medium contains 6 g l^{-1} Na_2HPO_4, 3.0 g l^{-1} KH_2PO_4, 1.0 g l^{-1} NH_4Cl, 0.5 g l^{-1} NaCl, 1 mM $MgSO_4$, 0.1 mM $CaCl_2$.

Analytical Methods

The concentrations of **1a-1c** and **2a-2c** was analysed by GC-MS: EtOAc extracts containing **1a-1c** and **2a-2c** were analysed using a Shimadzu GC-2010 Plus system with a GC-MS-QP2020 detector. Column: Agilent HP-5 (30 m × 0.25 mm × 0.25 μm). Temperature program: start at 40 °C, keep at for 1 min, then

increase to 280 °C at 20 °C min^{-1}, hold at 280 °C for 2 min. **1a-1c** and **2a-2c** were detected by total ion chromatogram and quantified by selective ion monitoring mode (SIM) with m/z = 67. Retention times: 1.4 min for **1a**; 2.1 min for **1b**; 2.9 min for **1c**; 2.1 min for **2a**; 2.9 min for **2b**; 3.8 min for **2c**. Acetophenone (~1 mM) was used as the internal standard for the GC-MS analysis (retention time 5.2 min). The GC-chromatograms and MS-spectra of **1a-1c** produced from various substrates are provided in Supplementary Figure 17-19.

GS-MS was also used to analyse **3a-3c**, **4a-4c**, **5**, and **6**. The analysis of acids (**3a-3c**, **4a-4c**, and **6**) requires first derivatization to the corresponding methyl esters before the analysis. 300 μl of dried EtOAc extracts containing targeted acids was mixed 180 μl of MeOH and 20 μl of (trimethylsilyl)diazomethane solution (2.0 M in diethyl ether) in GC vials. The mixtures were incubated at 25 °C, 300 rpm for 30 min. Then the GC vials were immediately analysed using a Shimadzu GC-2010 Plus system with a GCMS-QP2020 detector. Column: Agilent HP-5 (30 m × 0.25 mm × 0.25 μm). Temperature program: start at 40 °C, keep at for 1 min, then increase to 280 °C at 20 °C min^{-1}, hold at 280 °C for 2 min. **3a-3c**, **4a-4c**, **5**, and **6** were detected by total ion chromatogram and quantified by selective ion monitoring mode (SIM) with different m/z. Retention times (and characteristic m/z): 5.7 min for **3a** (124); 6.4 min for **3b** (138); 7.2 min for **3c** (152); 8.6 min for **4a** (185); 9.2 min for **4b** (199); 9.8 min for **4c** (213); 9.5 min for **5** (236); 11.6 min for **6** (264). Acetophenone (~1 mM) was used as the internal standard for the GC-MS analysis (retention time 5.2 min).

High-throughput UPLC-MS method was used to measure **4a**, **4b**, **6** and 9-hydroxylnonanoic acid during the screening of *E. coli* libraries for **4a** and **4b** production (Figure 6, Figure S12, S13). The acidified aqueous samples were analysed by using a Waters Acquity UPLC system. Column: Acquity uplc CSH C18 column (2.1 x 50mm, 1.7 um particles). Flow rate: keep constant at 0.61 ml min^{-1}. Flow program: 95% water (0.1% FA) and 5% ACN (0.1% FA) for 0-0.5 min, linear increase to 10% water (0.1% FA) and 90% ACN (0.1% FA) for 0.5-4 min, linear increase to 2% water (0.1% FA) and 98% ACN (0.1% FA) for 4-4.5 min, keep at 2% water (0.1% FA) and 98% ACN (0.1% FA) for 4.5-5 min, decrease to 95% water (0.1% FA) and 5% ACN (0.1% FA) for 5-6 min, and keep at 95% water (0.1% FA) and 5% ACN (0.1% FA) for 6-6.5 min. **4a**, **4b**, **6** and 9-hydroxylnonanoic acid were detected and quantified by single ion recording (negative mode) with different characteristic m/z. Retention times (and characteristic m/z): 2.13 min for **4a** (187); 2.37 min for **4b** (201); 4.8 min for **6** (281); 2.20 min for 9-hydroxylnonanoic acid (173). Benzyl alcohol (1 mM) was used as the internal standard for the UPLC-MS analysis (based on UV absorbance, retention time 1.65 min).

DNA Sequences

The OleT and related CamAB genes were kindly provided by Prof. K. Faber and Dr. A. Dennig from the University of Graz.[3]

The UndA gene from *Pseudomonas putida* F1 (Pput_3952)[4] was codon optimized for *E. coli* and synthesized by Gene Univeral on pET28a (NdeI/XhoI site). The optimized DNA sequence is:

>UndA

ATGGAAATCACCCGTATTAAAGAACTGAAAGTTATTGATGCCTTCGTTCGCATTGGCCCGCTGATGGACCCTGCAA
GTTATCCGCAGTGGGCACAGCAGCTGATTGAAGATTGCCGCGAAAGCAAACGTCGCGTTGTGGAACATGAATTTT
ATGCCCGCCTGCGTGATGGCCAGCTGAAACAGAGCACCATTCGCCAGTATCTGATTGGTGGCTGGCCGGTTGTGG
AACAGTTTAGTCTGTATATGGCACATAATCTGACCAAAACCCGTTATGGCCGCCATCAGGGCGAAGATATGGCACG
TCGCTGGCTGATGCGCAATATTCGCGTGGAACTGAATCATGCAGATTATTGGGTGAATTGGTGTCAGGCCCACGG
TGTTCATCTGCATGAACTGCAAGCCCAGGAAGTTCCGCCGGAACTGAATGGTCTGAATGATTGGTGCTGGCGCGT
GTGCGCAACCGAAAATCTGGCCATTAGCATGGCAGCCACCAATTATGCCATTGAAGGCGCCACCGGCGAATGGAG
TGCCGTGGTGTGTAGTACCGATACCTATGCACAGGGCTTTCCGGAAGAAGGTCGCAAACGTGCCATGAAATGGCT
GAAAATGCATGCCCAGTATGATGATGCCCATCCGTGGGAAGCACTGGAAATTATTTGTACCCTGGCAGGCGAAAA
TCCGACCCTGGGTCTGCGTACCGAACTGCGTCGCGCCATTTGCAAAAGCTATGATTGTATGTTTCTGTTTCTGGAAC
GTTGTATGCAGCTGGAAGGCCGTCAGCAGGGTCGCATGCGTCCGGCACTGGCCGCAGGTTAA

The UndB gene from *Pseudomonas mendocina* ymp (Pmen_4370)[5] was codon optimized for *E. coli* and synthesized by Gene Univeral on pET28a (NdeI/XhoI site). The optimized DNA sequence is:

>UndB

ATGAGCCCGAGCCCGGCTTCACTGAATGATCAGCAGCGCGCAGCACATATTCGTGAACAGGTTATGGCCCACGGT
AATGCACTGCGTCAGCGTTATCCGATTCTGCAACATCAGGATGCACTGGGCGCCGGCATTCTGGCCTTTGCACTGT
GTGGTATGATTGGCAGCGCAGCACTGTATATTGGTGGCCATCTGCCGTGGTGGGCATGTCTGCTGCTGAATGCCTT
TTTCGCAAGTCTGACCCATGAACTGGAACATGATCTGATTCATAGCATGTATTTTCGTAAACAGCCGCTGCCGCATA
ATCTGATGCTGGCACTGGTTTGGCTGGCCCGTCCGAGTACCATTAATCCGTGGGTGCGCCGTCATCTGCATCTGAA
TCATCATAAAGTGAGCGGTAGTGAAGCCGATATGGAAGAACGCGCCATTACCAATGGCGAACCGTGGGGTATTG
CCCGCCTGCTGATGGTTGGCGATAATATGATGAGTAGTTTTATTCGTTGGCTGCGTGCAAAAAATCCGGAACATCG
CCGCCTGATTCTGACCCGCACCCTGAAAGTGTATGCACCGCTGGGCCTGCTGAATTGGGCCACCTGGTATTTATTTC
TGGGTTTTCATCTGCTGGATTGGGCAGCCGCAGCACTGGGCGCGCCTATTGCATGGAGCGCAAGCACCCTGAGTG
TGATGCAGGTGGTGAATGTTGCCGTTGTTGTGCTGGTTGGCCCGAATGTGCTGCGCACCTTTTGTCTGCATTTTGT
GAGTAGCAATATGCATTATTACGGCGATGTTGAACCGGGTAATGTGATTCAGCAGACCCAGGTTCTGAATCCGTG
GTGGCTGTGGCCGCTGCAAGCATTTTGTTTTAATTTTGGTAGCAGTCATGCAATTCATCATTTTGTGGTTAAAGAAC
CGTTTTATATCCGCCAGCTGACCGTTCCGTTTGCACATCGTGTTATGCGTGAAATGGGTGTTCGCTTTAATGATTTT
GGTACATTTGCCCGTGCCAATCGCTGGACCCGCCGTGCTCGCACCCAGCAAGAACGTGCAAGCACCGCCTAA

The PfBVMO gene from *Pseudomonas fluorescens*[6] was codon optimized for *E. coli* and synthesized by Gene Univeral on pET28a (NdeI/XhoI site). The optimized DNA sequence is:

>PfBVMO

ATGAACGCCCATAGCGATAGCATTGATATTGCAATTATTGGCAGTGGCTTTGCAGGTCTGTGCATGGCCATTAAAC
TGAAAGAAGCCGGTTTTACCGATTTATTTGTTGCCGAACAGGCCGATACCCTGGGCGGTACATGGCGCGATAATC
ATTATCCGGGCTGCGCATGTGATGTTCAGAGTCATGTGTATAGCTTTAGCTTTGCACCGAATCCGGATTGGACCCG
CCAGTTTGCACCGCAGGCCGAAATTCGCGCCTATCTGGAAGATTGCGCCGTTCGCTTTGGCCTGGCACCGTATCTG
CGCTTTGGTATGGGTCTGAAACGCGCAGTTTTTGATGAACAGCTGCAACGCTGGCAGCTGAGTTTTAGTGATGGTC
GTCATGTGAGTGCCCGTGTGCTGGTTAGTGGTATGGGCGCCCTGGCACGTCCGGCACTGCCTGAAATTCCGGGCC
TGGAAACCTTTAAAGGTAAACGTTTTCATAGCCAGCAGTGGGATCATGCATACGCTCTGAAAGGTAAACGCGTTG
CAGTTATTGGCACCGGCGCAAGCGCAATTCAGTTTGTTCCGCAGATTGCCCCGCAGGTGGCCCATCTGGATTTATT
TCAGCGCACCCCGCCGTGGATTATGCCGAAACCGGATCGTGGTATTAGTGCCTTTGAACGTTGGCTGTTTCGTCAT
CTGCCGGTGACCCAGCGTCTGGTGCGCGGTGCTTTTTATTGGGCACTGGAAGGTCGTGTTCTGGGTTTTGCACTGC
ATCCGCAGCTGATGAAAATGGTTCAGAAAGTTGCACTGCGCCATCTGCGCAAACAGGTGCCGCGCCCGAGTCTGC
GTAAAGCCCTGACCCCGGATTATACCATTGGCTGCAAACGCGTGCTGATTAGTAATGATTATTATCCGGCCCTGAG
CCGTAGCAATGTTGAAGTTGTTACCGATAAAATTCTGCGCATTGAAGCAGATGGTGTTATTACCGCAGATGGCATT
AAACATCCGGCAGATTGCCTGATTTTTGGTACAGGTTTTCAGGCCACCGATCCGCTGCCGCGTGATTGTATTATTG
GTCGCGATGGTGTTGATCTGATGGATACCTGGCGCGATGGCGCCCATGCCTATAAAGGCACCACCGTGCCGGGTT
ATCCGAATTTATTTCTGATTATTGGTCCGAATACCGGCCTGGGTCATAATAGTATGATTCTGATGATTGAAGCACAG
GTGACCTATATTCTGGATGCACTGCGTCAGATGCAGCGCCATCGCATTGCCACCGTGGATGTTAAACCGATGGTGG
AACAGGCATATAATCGTCAGCTGCAAGATCAGCTGAAACGTACCATTTGGAATACCGGTGGTTGCCAGAGTTGGT
ATCTGGACCCTCGCACCGGCAAAAATACCACCCTGTGGCCGGCCAGTACCTGGCGCTTTAAACGTGTTACCCGTCA
GTTTGCCCTGAAAGATTATGCCGTGGATCTGCTGCCGCTGACCGCACCGCCGCGTCCTGCAACAGCACCGCATAGC
ACCGCCGAAGGTAGCCTGAGTTAA

The PpBVMO gene from *Pseudomonas putida*[7] was codon optimized for *E. coli* and synthesized by Gene Univeral on pET28a (NdeI/XhoI site). The optimized DNA sequence is:

>PpBVMO

ATGAGCAGTCATACCGCACTGCCGGTGGAACCGCTGGATGTTCTGATTATGGGCGCCGGTGTGAGCGGCATTGGT
GCAGCAGCCTATCTGCGCCGCAATCAGCCGAATAAGACCTTTGCCATTCTGGAAAGTCGTGAACGCATGGGTGGC
ACCTGGGATCTGTTTCGCTATCCGGGTATTCGTAGCGATAGCGATCTGTATACCTTTGGCTTTGATTTTAAACCGTG
GACCAAAGCCAAAAGTCTGGCCGATGCAGCCGATATTCTGGAATATCTGAGTGAAGCCATTGATGAACATCAGCT
GGCCCCGTTTATTCAGTATCAGCAGAAAGTTATTAGCGCCAATTGGCAGAGCGATAAAGGTCTGTGGAGCGTGCG
CGTTGAAGATGGCCGCACCGCCCAGATTCGTACCGTTGAATGTCGTTGGCTGTTTAGTGCCGGCGGCTATTATCGC
TATGATCAGGGCTTTAGCCCGCGTTTTGAAGGCAGCGAACAGTTTAAAGGCCAGATTATTCATCCGCAGCATTGGC
CGGAAGATTTGGATTATACCGGCAAACGTGTGGTTGTTATTGGCAGCGGTGCAACCGCAGTTACCCTGATTCCGG
CAATGGCTGATAAAGTTGCAAGCATTACCATGCTGCAACGTACCCCGAGTTATATTATTAATCAGCCGGCAAATGA
TGGCGTTGCCGCATTTCTGCGCAAAGTTCTGCCGGCCCAGACCGCATATAGCCTGACCCGTTATAAAAATGCAAAA
ATTACCCTGGCCTTTTGGGGTTTTTGCCAGCGCTTTCCGAAACTGAGCAAAAAACTGCTGCTGTGGCTGACCCGTA
AAGAACTGCCGAAAGATTATCCGGTGGATGTGCATTTTAATCCGCCGTATAATCCGTGGGATCAGCGCCTGTGTAG
CGTTCCGGAAGGCGATCTGTTTAAAGCAATTAGCGCCGGTAATGCCGATATTGTGACCGATCATATTGAACGTTTT
ACCGAACATGGCGTGCTGCTGAAAAGTGGCAAAATGCTGAAAGCAGATATTATTGTTACCGCCACCGGCCTGAAT
GTGCAGCTGTTTGGTGGCATTACCCTGCATAAAGATGGCAAACCGGTTGTTCTGAGCGAAACCCTGGCCTATAAA
GGTATGATGCTGAGTGGCGTGCCGAATTTTGCCTTTGCAGTTGGCTATACCAATAGCAGCTGGACCCTGAAAGTGT

GTCTGCTGTGCGATCATTTTTGTCGCCTGCTGGGCCTGATGGAACGTGAAGGTTATAATGTGTGTGAACCGAAAGC
ACCGGAAGGCGTGGAAACCCGTCCGCTGCTGGATTTTGGTGCAGGTTATGTTCAGCGTGCACTGGATAGCATGCC
GCGTCAGGGCCCGCGCGAACCTTGGGTTATGAGTATGGATTATTTTCGCGATGTTAAACTGCTGCGTCGCGGCGC
CGTGACCGATAAATGTCTGAAATTCACTGCCGTTCCGAATGCACCGCTGCATGCCGATGTTCAGCTGCAACAGCAG
GGTAGCCGTCGTTAA

The MlADH gene from *Micrococcus luteus*[1] was codon optimized for *E. coli* and synthesized by Gene Univeral on pET28a (NdeI/XhoI site). The optimized DNA sequence is:

>MlADH

ATGAGCGAATTTACCCGTTTTGAACAGGTGACCGTGCTGGGTACAGGTGTGCTGGGCAGTCAGATTATTATGCAG
GCAGCCTATCATGGCAAAAAAGTTATGGCCTATGATGCCGTGCCGGCAGCCCTGGAAAATCTGGATAAACGCTGG
GCATGGATTCGTCAGGGCTATGAAGCCGATCTGGGCGAAGGTTATGATGCCGCCCGTTTTGATGAAGCCATTGCC
CGCATTACCCCGACCAGTGATCTGGCAGAAGCCGTGGCAGATGCAGATATTGTTATTGAAGCAGTTCCGGAAAAT
CTGGAACTGAAACGCAAAGTTTGGGCACAGGTGGGTGAACTGGCCCCGGCCACCACCCTGTTTGCCACCAATACC
AGCAGTCTGCTGCCGAGTGATTTTGCCGATGCAAGCGGCCATCCGGAACGCTTTCTGGCACTGCATTATGCAAATC
GCATTTGGGCACAGAATACCGCCGAAGTTATGGGTACAGCAGCAACCAGTCCGGAAGCCGTGGCGGGCGCACTG
CAATTTGCAGAAGAAACCGGTATGGTGCCGGTGCATGTTCGCAAAGAAATTCCGGGTTATTTTCTGAATAGCCTGC
TGATTCCGTGGCTGCAAGCCGGTAGCAAACTGTATATGCATGGCGTTGGTAATCCGGCAGATATTGATCGCACCTG
GCGCGTTGCCACCGGCAATGAACGTGGTCCGTTTCAGACCTATGATATTGTGGGCTTTCATGTGGCAGCCAATGTT
AGTCGTAATACCGGTGTGGATTGGCAGCTGGGCTTTGCCGAAATGCTGGAAAAAGCATTGCCGAAGGCCATAGC
GGCGTTGCCGATGGCCAGGGTTTTTATCGCTATGGCCCGGATGGTGAAAATCTGGGTCCGGTTGAAGATTGGAAT
CTGGGTGATAAAGATACCCCGCTGGGTTAA

The OhyA2 gene from *Stenotrophomonas maltophilia*[8] was codon optimized for *E. coli* and synthesized by Gene Univeral on pET28a (NdeI/XhoI site). The optimized DNA sequence is:

>OhyA2

ATGAGTCAGCCGACCGCCCCGGGCCGTAATGCCGGTGCAACCCCTGCCTTTGAACATGAACCGGATAGCACCGGC
GGCTATTGGAGCAATCGTCCGGAAAATACCCTGCCGCCGCCGGATATGATGGGCGCCTATATGCGTAATCGTCCG
CTGCCGCCGGAAGATGTTGCCCAGCGTAAAGCATATATTATTGGTACAGGTATTGCCGGTCTGGCAGCAGCATTTT
ATCTGATTCGTGATGGTGGTATGCCGCCGGCCAATATTACCCTGCTGGATAGTCTGGAAATTGAAGGTGGCAGTCT
GGATGGTGCAGGTGATGCAGAACAGGGTTATCTGATTCGCGGTGGCCGTGAAATGAATTGGAATTATGATAATTT
CTGGGACCTGTTTCAGGATGTGCCGGCCCTGGAACTGCCGGCCGGTTTTAGTGTGCTGGATGAATATCGCGCAGT
GAATGATAATGATCCGAATTGGAGCAAAGCACGCCTGCTGCATCAGCAGGGCAAAGTTAAAGATTTTGCCACCTTT
GGTCTGAGCCGTGGTCAGCAGTGGGAACTGGTGAAACTGCTGCTGAAACGCAAAGAAGATTTAGATGATGTGAC
CATTGAAGATTATTTCAGCGAAGGTTTTCTGCAAAGCAATTTTTGGTTTTTCTGGCGCAGCATGTTTGCATTTGAAA
ATTGGCAGAGTCTGCTGGAAATGAAACTGTATATGCATCGTTTTCTGGATGCAATTGATGGCCTGAATGATATGAG
CGCACTGGTGTTTCCGAAATATAATCAGTATGAAAGTTTCGTGGTGCCGCTGAGTCGCATGCTGCGTGCCCAGGGT
GTGAATGTGCAGTTTGATACCCGTGTGCATGATCTGGAAATGGCCGTTGATGGCCAGAGTCGCACCGTGACCGCC
CTGCGTTGCCGTGTGGCAGGCAATGAAACCACCCTGCCGGTGGCCGCCGGTGATCTGGTTTTTGCACTGACCGGC
AGCATGACCGAAGGTACAGCCTATGGTGATATGGATACCGTTCCGCCGCTGGCACGTGATCGCCGCGATCCTGGT

GAAGATAGTGATTGGGCACTGTGGCGTAATCTGGCCCGTCAGAGCCCGATTTTTGGCAAACCGGAAAAATTTTAT
GGCGATGTGGATCGCAGTATGTGGGAAAGTGCCACCCTGACCTGTCGCCCGAGCCCGTTAGTGGATAAAATTCGC
ACCCTGAGCGTTAATGATCCGTATAGTGGTCGTACCGTTACCGGTGGCGTTATTACCATTACCGATAGCAATTGGG
TGCTGAGCTTTACCGTGAATCGTCAGCCGCATTTTGTTGATCAGCCGAAAGATGTTCTGGTGGTTTGGGTTTATGC
CCTGCTGATGGATCAGGATGGTAATCATATTAAAAAGCCGATGCCGGCATGCACCGGCCGTGAAGTTCTGGCCGA
ACTGTGCCATCATCTGGGCATTGGCGATCAGATTGATGCAGTTGCAGCAGCCACCCGTGTTCGCCTGGCACTGATG
CCGTATATTACCGCCCAGTTTATGCCGCGTGCAGCAGGTGATCGTCCGCATGTTGTGCCGGCAGGCTGTACCAATC
TGGGTCTGCTGGGCCAGTTTGTTGAAACCCGTAATGATGTTATTTTCACAATGGAAAGCAGTATTCGTACCGCCCG
CGTTGCAGTGTATACCCTGCTGGGTCTGCGTAAACAGGTGCCGGATCTGAGTCCGACCCAGTATGATATTCGCAAT
CTGATTAAAGCCGCCCGCGCCCTGAATAATAATGCCCCGTTTCCGGGTGAACGTCTGCTGCATCGTCTGCTGGGCA
ATAGCTATTATGCCCATATTCTGCCGCCGCTGCCGCAGCCGGAAAAAGGCCGTGAAGCATTTCTGGAAGAAGAAC
TGAGTTGGCTGAGCGGTAAAGGTAGCGTGGTGCTGAAAGATTTAAGCGCCCGTCTGGATCGCCTGGGCGAAACC
CTGGGTCGCCGTCGTGCCCCTTAA

The TLL gene from *Thermomyces lanuginosus*[9] was codon optimized for *E. coli* and synthesized by Gene Univeral on pET28a (NdeI/XhoI site). The optimized DNA sequence is:

>TLL

ATGAGTCCTATTCGTCGAGAGGTCTCGCAGGATCTGTTTAACCAGTTCAATCTCTTTGCACAGTATTCCGCAGCCGC
ATACTGCGGAAAAAACAATGATGCCCCAGCTGGTACAAACATTACGTGCACGGGAAATGCCTGCCCCGAGGTAGA
GAAGGCGGATGCAACGTTTCTCTACTCGTTTGAAGACTCTGGAGTGGGCGATGTCACCGGCTTCCTTGCTCTCGAC
AACACGAACAAATTGATCGTCCTCTCTTTCCGTGGCTCTCGTTCCATAGAGAACTGGATCGGGAATCTTAACTTCGA
CTTGAAAGAAATAAATGACATTTGCTCCGGCTGTAGGGGACATGACGGCTTCACTTCGTCCTGGAGGTCTGTAGCC
GATACGTTAAGGCAGAAGGTGGAGGATGCTGTGAGGGAGCATCCCGACTATCGCGTGGTGTTTACCGGACATAG
CTTGGGTGGTGCATTGGCAACTGTTGCCGGAGCAGACCTGCGTGGAAATGGGTATGATATCGACGTGTTTTCATA
CGGCGCCCCCCGAGTCGGAAACAGGGCTTTTGCAGAATTTCTGACCGTACAGACCGGCGGAACACTCTACCGCAT
TACCCACACCAATGATATTGTCCCTAGACTCCCGCCGCGCGAATTTGGTTACAGCCATTCTAGCCCAGAGTACTGG
ATCAAATCTGGAACCCTTGTCCCCGTCACCCGAAACGATATCGTGAAGATAGAAGGCATCGATGCCACCGGCGGC
AATAACCAGCCTAACATTCCGGATATCCCTGCGCACCTATGGTACTTCGGGTTAATTGGGACATGTCTTTAA

The ChnD and ChnE genes from *Acinetobacter sp.* NCIMB9871[10] were codon optimized for *E. coli* and synthesized by Gene Univeral on pUC57. The optimized DNA sequences are:

>ChnD

ATGCATTGCTATTGCGTTACCCATCATGGTCAGCCGCTGGAAGATGTTGAAAAAGAAATTCCGCAGCCGAAAGGC
ACCGAAGTTCTGCTGCATGTTAAAGCAGCCGGTCTGTGCCATACCGATCTGCATCTGTGGGAAGGCTATTATGATC
TGGGTGGCGGCAAACGCCTGAGTCTGGCAGATCGTGGTCTGAAACCGCCGCTGACCCTGAGTCACGAAATTACCG
GTCAGGTTGTTGCCGTTGGCCCGGATGCCGAAAGCGTGAAAGTTGGCATGGTGAGCCTGGTGCATCCGTGGATTG
GCTGCGGTGAATGCAATTATTGCAAACGTGGCGAAGAAAATCTGTGCGCCAAACCGCAGCAGCTGGGCATTGCAA
AACCGGGCGGCTTTGCAGAATATATTATTGTTCCGCATCCGCGCTATCTGGTGGATATTGCAGGCCTGGATCTGGC
CGAAGCCGCACCGCTGGCATGTGCCGGTGTTACCACCTATAGCGCCCTGAAAAAATTTGGTGACCTGATTCAGAG
CGAACCGGTTGTTATTATTGGCGCAGGTGGCCTGGGTCTGATGGCACTGGAACTGCTGAAAGCAATGCAGGCAAA

AGGTGCAATTGTGGTTGATATTGATGATAGTAAACTGGAAGCAGCCCGCGCAGCAGGTGCACTGAGTGTGATTAA
TAGTCGTAGTGAAGATGCCGCACAGCAGCTGATTCAGGCAACCGATGGTGGCGCACGTCTGATTCTGGATCTGGT
TGGCAGTAATCCGACCCTGAGTCTGGCGCTGGCCAGCGCAGCCCGTGGTGGACATATTGTGATTTGTGGTCTGAT
GGGTGGCGAAATTAAGCTGAGCATTCCGGTTATTCCGATGCGCCCGCTGACCATTCAGGGCAGCTATGTTGGCAC
CGTTGAAGAACTGCGCGAACTGGTTGAACTGGTGAAAGAAACCCACATGAGCGCCATTCCGGTTAAAAAACTGCC
GATTAGTCAGATTAATAGTGCATTTGGTGACTTAAAAGACGGCAATGTGATTGGTCGCATTGTTCTGATGCATGAA
AATTAA

>ChnE

ATGAACTACCCGAATATTCCGCTGTATATTAATGGTGAATTTCTGGATCATACCAATCGTGATGTTAAAGAAGTGTT
TAATCCGGTGAATCACGAATGCATTGGTCTGATGGCATGTGCAAGCCAGGCCGATCTGGATTATGCCCTGGAAAG
TAGTCAGCAGGCCTTTCTGCGCTGGAAAAAGACTAGCCCGATTACCCGTAGTGAAATTCTGCGCACCTTTGCAAAA
CTGGCACGCGAAAAAGCAGCAGAAATTGGTCGCAATATTACCCTGGATCAGGGTAAACCGCTGAAAGAAGCAATT
GCAGAAGTGACCGTGTGTGCAGAACATGCCGAATGGCATGCAGAAGAATGCCGCCGTATCTATGGTCGTGTTATT
CCGCCGCGTAATCCGAATGTGCAGCAGCTGGTTGTGCGTGAACCGCTGGGTGTTTGCCTGGCATTTTCACCGTGG
AATTTTCCGTTTAATCAGGCAATTCGTAAAATTAGCGCCGCAATTGCCGCAGGCTGTACCATTATTGTGAAAGGCA
GCGGCGATACCCCGAGTGCCGTGTATGCAATTGCACAGCTGTTTCACGAAGCCGGTCTGCCGAATGGTGTTCTGA
ATGTTATTTGGGGTGACAGCAATTTTATTAGCGATTATATGATCAAGAGCCCGATTATTCAGAAAATTAGTTTTACC
GGCAGCACCCCGGTTGGCAAAAAACTGGCCAGCCAGGCCAGTCTGTATATGAAACCGTGCACAATGGAACTGGG
CGGCCATGCACCGGTTATTGTGTGCGATGATGCCGATATTGATGCAGCAGTGGAACATCTGGTGGGCTATAAATTT
CGCAATGCAGGTCAGGTGTGTGTGAGCCCGACCCGTTTTTATGTGCAGGAAGGTATCTATAAAGAATTTTCTGAAA
AGGTTGTTCTGCGTGCAAAACAGATTAAGGTGGGCTGCGGTCTGGATGCCAGCAGCGATATGGGTCCGCTGGCCC
AGGCCCGCCGTATGCATGCAATGCAGCAGATTGTGGAAGATGCAGTGCATAAAGGCAGTAAACTGCTGCTGGGC
GGCAATAAGATTAGTGATAAAGGCAATTTCTTTGAGCCGACCGTTCTGGGTGACCTGTGCAATGATACCCAGTTTA
TGAATGATGAACCGTTTGGTCCGATTATTGGTCTGATTCCGTTTGATACCATTGATCATGTTCTGGAAGAAGCAAAT
CGTCTGCCGTTTGGCCTGGCAAGTTATGCCTTTACCACCAGCAGCAAAAATGCCCATCAGATTAGTTATGGCCTGG
AAGCAGGCATGGTTAGCATTAATCACATGGGCCTGGCCCTGGCAGAAACCCCGTTTGGTGGCATTAAGGATAGCG
GTTTTGGTAGCGAAGGCGGCATTGAAACCTTTGATGGTTATCTGCGCACCAAATTCATTACCCAGCTGAATTAA

Genetic Engineering of *E. coli* Expressing a Single Enzyme

E. coli (OleT) expressing OleT and the putidaredoxin CamAB was engineered by transformation of *E. coli* BL21 (DE3) competent cells with pET28a-OleT[3] and pACYC-CamAB[3] together.

E. coli (UndA) expressing UndA was engineered by the following procedure. UndA was amplified from pET28a-UndA by using primers UndA-NcoI-F and pET28a-XhoI-R with Q5 DNA polymerase. The PCR product was double digested with fast digest enzymes NcoI and XhoI, and then ligated to the NcoI/XhoI, digested pRSFduet-1 with T4 DNA ligase. *E. coli* BL21 (DE3) competent cells was transformed with the ligation product, pRSF-UndA, to give *E. coli* (UndA).

E. coli (UndB) expressing UndB was engineered by the following procedure. UndB was amplified from pET28a-UndB by using primers UndB-BspHI-F and pET28a-XhoI-R with Q5 DNA polymerase. The PCR product was double digested with fast digest enzymes BspHI and XhoI, and then ligated to the NcoI/XhoI digested pRSFduet-1 with T4 DNA ligase. *E. coli* BL21 (DE3) competent cells was transformed with the ligation product, pRSF-UndB, to give *E. coli* (UndB).

E. coli (TLL) expressing TLL was engineered by the following procedure. TLL was amplified from pET28a-TLL by using primers TLL-BspHI-F and TLL-XhoI-R with Q5 DNA polymerase. The PCR product was double digested with fast digest enzymes BspHI and XhoI, and then ligated to the NcoI/XhoI digested pRSFduet-1 with T4 DNA ligase. *E. coli* BL21 (DE3) competent cells was transformed with the ligation product, pRSF-TLL, to give *E. coli* (TLL).

E. coli (FadL) expressing FadL was engineered by the following procedure. FadL was amplified from the genome of *E. coli* BL21 (DE3) by using primers FadL-BspHI-F and FadL-XhoI-R with Q5 DNA polymerase. The PCR product was double digested with fast digest enzymes BspHI and XhoI, and then ligated to the NcoI/XhoI digested pACYCduet-1 with T4 DNA ligase. *E. coli* BL21 (DE3) competent cells was transformed with the ligation product, pACYC-FadL, to give *E. coli* (FadL).

Genetic Engineering of Different Tagged PfBVMOs and PpBVMOs

PfBVMO was engineered with different N-terminal tags or fusion proteins. They were assembled using the following procedures.

E. coli (PfBVMO) expressing PfBVMO was engineered by the following procedure. PfBVMO was amplified from pET28a-PfBVMO by using primers PfBVMO-BspHI-F and pET28a-XhoI-R with Q5 DNA polymerase. The PCR product was double digested with fast digest enzymes BspHI and XhoI, and then ligated to the NcoI/XhoI digested pRSFduet-1 with T4 DNA ligase. *E. coli* BL21 (DE3) competent cells was transformed with the ligation product, pRSF-PfBVMO, to give *E. coli* (PfBVMO).

E. coli (E6-PfBVMO) expressing E6-PfBVMO was engineered by the following procedure. E6-PfBVMO was amplified from pET28a-PfBVMO by using primers PfBVMO-NcoI-E6-F and pET28a-XhoI-R with Q5 DNA polymerase. The PCR product was double digested with fast digest enzymes NcoI and XhoI, and then ligated to the NcoI/XhoI digested pRSFduet-1 with T4 DNA ligase. *E. coli* BL21 (DE3) competent cells was transformed with the ligation product, pRSF-E6-PfBVMO, to give *E. coli* (E6-PfBVMO).

E. coli (H6-PfBVMO) expressing H6-PfBVMO was engineered by the following procedure. H6-PfBVMO was cut from pET28a-PfBVMO by fast digest enzymes NcoI and XhoI, and then ligated to the NcoI/XhoI

digested pRSFduet-1 with T4 DNA ligase. *E. coli* BL21 (DE3) competent cells was transformed with the ligation product, pRSF-H6-PfBVMO, to give *E. coli* (H6-PfBVMO).

E. coli (K6-PfBVMO) expressing E6-PfBVMO was engineered by the following procedure. K6-PfBVMO was amplified from pET28a-PfBVMO by using primers PfBVMO-BspHI-K6-F and pET28a-XhoI-R with Q5 DNA polymerase. The PCR product was double digested with fast digest enzymes BspHI and XhoI, and then ligated to the NcoI/XhoI digested pRSFduet-1 with T4 DNA ligase. *E. coli* BL21 (DE3) competent cells was transformed with the ligation product, pRSF-K6-PfBVMO, to afford *E. coli* (K6-PfBVMO).

E. coli (MBP-PfBVMO) expressing MBP-PfBVMO was engineered by the following procedure. MBP was amplified from the genome of *E. coli* BL21 (DE3) using primers MBP-BspHI-F and MBP-NdeI-R with Q5 DNA polymerase. The PCR product was double digested with fast digest enzymes BspHI and NdeI, and then ligated to the NcoI/NdeI digested pRSFduet-1 to give pRSF-MBP. PfBVMO was cut from pET28a-PfBVMO by fast digest enzymes NdeI and XhoI, and then ligated to the NdeI/XhoI digested pRSF-MBP with T4 DNA ligase. *E. coli* BL21 (DE3) competent cells was transformed with the ligation product, pRSF-MBP-PfBVMO, to afford *E. coli* (MBP-PfBVMO).

E. coli (Trx-PfBVMO) expressing Trx-PfBVMO was engineered by the following procedure. Trx was amplified from the genome of *E. coli* BL21 (DE3) using primers Trx-BspHI-F and Trx-NdeI-R with Q5 DNA polymerase. The PCR product was double digested with fast digest enzymes BspHI and NdeI, and then ligated to the NcoI/NdeI digested pRSFduet-1 to give pRSF-Trx. PfBVMO was cut from pET28a-PfBVMO by fast digest enzymes NdeI and XhoI, and then ligated to the NdeI/XhoI digested pRSF-Trx with T4 DNA ligase. *E. coli* BL21 (DE3) competent cells was transformed with the ligation product, pRSF-Trx-PfBVMO, to afford *E. coli* (Trx-PfBVMO).

E. coli (TrxGS-PfBVMO) expressing TrxGS-PfBVMO was engineered by the following procedure. GS-PfBVMO was amplified from pET28a-PfBVMO by using primers PfBVMO-NdeI-GS-F and pET28a-XhoI-R with Q5 DNA polymerase. The PCR product was double digested with fast digest enzymes NdeI and XhoI, and then ligated to the NdeI/XhoI digested pRSF-Trx with T4 DNA ligase. *E. coli* BL21 (DE3) competent cells was transformed with the ligation product, pRSF-TrxGS-PfBVMO, to afford *E. coli* (TrxGS-PfBVMO).

PpBVMO was also engineered with different N-terminal tags or fusion proteins. They were assembled using the following procedures.

E. coli (PpBVMO) expressing PpBVMO was engineered by the following procedure. PpBVMO was amplified from pET28a-PpBVMO by using primers PpBVMO-BspHI-F and pET28a-XhoI-R with Q5 DNA polymerase. The PCR product was double digested with fast digest enzymes BspHI and XhoI, and then ligated to the NcoI/XhoI digested pRSFduet-1 with T4 DNA ligase. *E. coli* BL21 (DE3) competent cells was transformed with the ligation product, pRSF-PpBVMO, to afford *E. coli* (PpBVMO).

E. coli (E6-PpBVMO) expressing E6-PpBVMO was engineered by the following procedure. E6-PpBVMO was amplified from pET28a-PpBVMO by using primers PpBVMO-NcoI-E6-F and pET28a-XhoI-R with Q5 DNA polymerase. The PCR product was double digested with fast digest enzymes NcoI and XhoI, and then ligated to the NcoI/XhoI digested pRSFduet-1 with T4 DNA ligase. *E. coli* BL21 (DE3) competent cells was transformed with the ligation product, pRSF-E6-PpBVMO, to afford *E. coli* (E6-PpBVMO).

E. coli (H6-PpBVMO) expressing H6-PpBVMO was engineered by the following procedure. H6-PpBVMO was cut from pET28a-PpBVMO by fast digest enzymes NcoI and XhoI, and then ligated to the NcoI/XhoI digested pRSFduet-1 with T4 DNA ligase. *E. coli* BL21 (DE3) competent cells was transformed with the ligation product, pRSF-H6-PpBVMO, to afford *E. coli* (H6-PpBVMO).

E. coli (K6-PpBVMO) expressing E6-PpBVMO was engineered by the following procedure. K6-PpBVMO was amplified from pET28a-PpBVMO by using primers PpBVMO-BspHI-K6-F and pET28a-XhoI-R with Q5 DNA polymerase. The PCR product was double digested with fast digest enzymes BspHI and XhoI, and then ligated to the NcoI/XhoI digested pRSFduet-1 with T4 DNA ligase. *E. coli* BL21 (DE3) competent cells was transformed with the ligation product, pRSF-K6-PpBVMO, to give *E. coli* (K6-PpBVMO).

E. coli (NusA-PpBVMO) expressing NusA-PpBVMO was engineered by the following procedure. NusA was amplified from the genome of *E. coli* BL21 (DE3) by using primers NusA-BspHI-F and NusA-NdeI-R with Q5 DNA polymerase. The PCR product was double digested with fast digest enzymes BspHI and NdeI, and then ligated to the NcoI/NdeI digested pRSFduet-1 to give pRSF-NusA. PpBVMO was cut from pET28a-PpBVMO by fast digest enzymes NdeI and XhoI, and then ligated to the NdeI/XhoI digested pRSF-NusA with T4 DNA ligase. *E. coli* BL21 (DE3) competent cells was transformed with the ligation product, pRSF-NusA-PpBVMO, to afford *E. coli* (NusA-PpBVMO).

E. coli (TrxGS-PpBVMO) expressing TrxGS-PpBVMO was engineered by the following procedure. GS-PpBVMO was amplified from pET28a-PpBVMO by using primers PpBVMO-NdeI-GS-F and pET28a-XhoI-R with Q5 DNA polymerase. The PCR product was double digested with fast digest enzymes NdeI and XhoI, and then ligated to the NdeI/XhoI digested pRSF-Trx with T4 DNA ligase. *E. coli* BL21 (DE3) competent cells was transformed with the ligation product, pRSF-TrxGS-PpBVMO, to give *E. coli* (TrxGS-PpBVMO).

Engineering of *E. coli* with Combinatorial RBS Libraries

The RBS Calculator (https://salislab.net/software/)[11,12] in the 'Design: RBS Sequences' mode was used to generate context-specific RBSs with a target translation initiation rate of 100,000 for each gene in its specific genetic context within the operons. The resulting synthetic RBSs were used as a starting point to generate RBS prediction data using the RBS Library Calculator[11,13] in the 'Predict: RBS Library' mode. For this, the eight base positions with a high impact on TIR were selected and fully randomized with degenerate bases (8N). These 8N libraries containing 65,536 sequence-TIR pairs for each gene were used as input for the RedLibs algorithm[14]. The target library size was set to four and the target library distribution was set to a uniform distribution between the minimum and maximum TIR values for each gene's 8N library. The resulting partially degenerate sequences coding for close-to-uniformly distributed TIR values (Supplementary Figure 20) were used to design primers for library construction (Supplementary Table 1).

The *E. coli* library for co-expressing E6-PfBVMO, OhyA2, MlADH, TLL, and FadL to produce **4b** was constructed on two plasmids pRSF-PfBVMO-OhyA2-MlADH-TLL and pACYC-FadL. The library for co-expressing H6-PpBVMO, OhyA2, MlADH, TLL, and FadL to produce 9-hydroxynonanoic acid was constructed on two plasmids pRSF-PpBVMO-MlADH-OhyA2-TLL and pACYC-FadL. The library for co-expressing H6-PpBVMO, OhyA2, MlADH, TLL, ChnD, ChnE and FadL to produce **4a** was constructed using the optimized pRSF-PpBVMO-MlADH-OhyA2-TLL and pACYC-ChnD-ChnE-FadL.

All amplifications of DNA fragments were performed by PCR using the Q5 DNA polymerase (New England Biolabs). The gene fragments for operon library construction were amplified with primers containing the corresponding degenerate RBS in a primer overhang. Primers RBS4-PfBVMO-F and PfBVMO-RBS4-R were used to amplify E6-PfBVMO from pRSF-E6-PfBVMO. Primers RBS4-MlADH-F and MlADH-RBS4-R were used to amplify MlADH from pET28a-MlADH. Primers RBS4-OhyA-F and OhyA-RBS4-R were used to amplify OhyA2 from pET28a-OhyA2. Primers RBS4-TLL-F and pRSF-RBS4-R were used to amplify pRSF-TLL from pRSF-TLL. These four fragments containing the degenerate RBSs were assembled to generate the operon PfBVMO-MlADH-OhyA2-TLL on a pRSFduet-1 plasmid backbone using the NEBuilder HiFi DNA assembly master mix (New England Biolabs) and transformed into electrocompetent cells of *E. coli* (FadL) containing pACYC-FadL.

Primers PpBVMO-RBS4-F and PpBVMO-RBS4-R were used to amplify H6-PpBVMO from pRSF-H6-PpBVMO. Primers RBS4-MlADH-F2 and MlADH-RBS4-R were used to amplify MlADH from pET28a-MlADH. Primers RBS4-OhyA-F and OhyA-RBS4-R were used to amplify OhyA2 from pET28a-OhyA2. Primers RBS4-TLL-F and pRSF-RBS4-R2 were used to amplify pRSF-TLL from pRSF-TLL. These four

fragments containing the degenerate RBSs were assembled to generate the operon PpBVMO-OhyA2-MlADH-TLL on a pRSFduet-1 plasmid backbone using the NEBuilder HiFi DNA assembly master mix and transformed into electrocompetent cells of *E. coli* (FadL) containing pACYC-FadL. The optimized pRSF-PpBVMO-MlADH-OhyA2- TLL was isolated from the best strain P11-F9 (Supplementary Figure 12).

Primers ChnD-RBS4-F and RBS4-ChnD-R were used to amplify ChnD from pUC57-ChnD. Primers ChnE-RBS4-F and RBS4-ChnE-R were used to amplify ChnE from pUC57-ChnE. Primers FadL-RBS4-F and pACYC-RBS4-R were used to amplify pACYC-FadL from pACYC-FadL. These three fragments containing the degenerate RBSs were assembled to generate the operon ChnD-ChnE-FadL on a pACYCduet-1 plasmid backbone using the NEBuilder HiFi DNA assembly master mix and transformed into electrocompetent cells of *E. coli* containing pRSF-PpBVMO-MlADH-OhyA2-TLL.

Screening of *E. coli* Libraries for production of 4b and 4a

To identify by screening the most effective *E. coli* strain that produces **4b**, LB (300 µl, containing 50 mg l^{-1} of kanamycin and 50 mg l^{-1} of chloramphenicol) in six deep 96-well plates was inoculated with the strain library described in 3.6. The cells were cultured for 8 h at 37 °C, 300 rpm. Then 100 µl of the cell culture were used to make cell stocks and stored in a deep freezer. To the rest of cell culture in each well, M9 medium (700 µl, with 2% glucose, 0.6% yeast extract, 50 mg l^{-1} of kanamycin, 50 mg l^{-1} of chloramphenicol) and IPTG (final concentration 0.5 mM) were added. The cells were then cultured at 22 °C, 300 rpm for 12-14 h. The cells were harvested by centrifugation (4000 g, 5 min) and resuspended in KP buffer (300 µl, 200 mM, pH 8.0, 1% glucose). **6** stock solution (7.5 µl, 200 mM in EtOH) were added into each well. And the reaction of **6** to **4b** was performed at 30 °C 300 rpm for 24 h. After reaction, the 96-well plates were subjected to centrifugation (4000 g, 15 min) and supernatant (100 µl) was collected and mixed with formic acid solution (900 µl, 0.3%) containing benzyl alcohol (1 mM). The mixture was subjected to centrifugation (4000 g, 15 min) and the supernatant (400 µl) was transferred into a new 96-well plate for high-throughput UPLC-MS analysis of **4b**.

LB medium (1 ml, with 50 mg l^{-1} of kanamycin and 50 mg l^{-1} of chloramphenicol) was inoculated with the best 4 strains for **4b** production (from each 96-well plate) for 8 h at 37 °C 250 rpm. The cultures were transferred to modified M9 medium (50 ml, with 2% glucose and 0.6% yeast extract) in baffled flasks to grow at 37 °C until OD_{600} of the culture reached 0.6-0.8. At this time, IPTG was added to a final concentration of 0.5 mM. The culture was carried on at 22 °C for 12-14 h. The *E. coli* cells were harvested by centrifugation and resuspended in KP buffer (200 mM, pH 8.0). UV-spectrometry was used to determine

the density of cells (OD_{600}). To an air-tight reaction tube (25 ml) with a screw-cap, a stock solution of cells, KP buffer (200 mM, pH 8.0) and a stock solution of glucose (50%) were added to afford a catalytic system (0.5 ml) containing with cells (10 g l^{-1}) and glucose (1%). A stock solution (12.5 µl) containing oleic acid (**6**) in EtOH (200 mM) was added to initiate the reaction (at 250 rpm, 30 °C for 24 h). Upon completion, the reaction mixtures were acidified with HCl (25 µl, 10 M) and saturated with NaCl. EtOAc (1 ml, containing 1 mM of acetophenone as internal standard) was added to extract **4b**. The EtOAc containing **4b** was dried over Na_2SO_4, derivatized and analysed by GC-MS.

The same procedure was applied to screening the most effective *E. coli* cell to produce 9-hydroxynonanoic acid and **4a** from **6**.

Procedure for Decarboxylation of 4a-4c and 3a-3c

4a-4c and **3a-3c** were dissolved in EtOH to prepare the substrate stock solutions (250 mM). *E. coli* (OleT), *E. coli* (UndA), and *E. coli* (UndB) were cultured and harvested using standard procedure. Freshly harvested cells were resuspended in KP buffer (200 mM, pH 8.0) to prepare the stock solutions of cells. The density of cells (OD_{600}) was determined with a UV-spectrometer. To an air-tight reaction tube (25 ml) with screw-cap, stock solutions of cells, KP buffer (200 mM, pH 8.0) and stock solution of glucose (50%) were added to form a catalytic system (0.5 ml) containing cells (10 g l^{-1}) and glucose (1-2%). Stock solutions of **4a-4c** and **3a-3c** (10 µl) were added to the reaction tubes. The reaction tubes were sealed and incubated at 250 rpm, 30 °C for 24 h. Upon completion, the reaction tubes were incubated on ice for 15-20 min, prior to opening these (to minimize the loss of volatile alkenes) and adding of EtOAc (1 ml, containing 1 mM of acetophenone as internal standard). The **2a-2c** were extracted, dried over Na_2SO_4 and analysed by GC-MS.

Procedure for Metathesis of 2a-2c

2a-2c were dissolved in EtOH to prepare the substrate stock solutions (250 mM). **Ru1**, **Ru2**, and **Ru3** were freshly dissolved in DSMO: EtOH = 1: 1 to prepare the stock solution (5 mM). To an GC vial (1.5 ml) with screw-cap, KP buffer (200 mM, pH 8.0) and stock solution of Ru-catalyst (optional: 5% TPGS-750-M in KP buffer) were added to form a system (100 µl) with Ru catalyst (100-250 µM). Optionally, *n*-dodecane (10 µl) was added. Then, the stock solutions of **2a-2c** (2 µl) were added to the reaction vials and the vials were incubated at 300 rpm, 30 °C for 24 h. Upon completion, the reaction vials were incubated on ice for 15-20 min prior to opening these (to minimize the loss of volatile alkenes), and EtOAc was added (1 ml,

containing 1 mM of acetophenone as internal standard). The **1a-1c** and **2a-2c** were extracted, dried over Na_2SO_4 and analysed by GC-MS.

Procedure for Chemoenzymatic Conversion of 4a-4c to 1a-1c

Freshly harvested *E. coli* (UndB) cells were resuspended in KP buffer (200 mM, pH 8.0) to prepare the stock solutions of cells. The density of cells (OD_{600}) was determined with a UV-spectrometer. To an air-tight reaction tube (25 ml) with screw-cap, stock solutions of cells, KP buffer (200 mM, pH 8.0) and stock solution of glucose (50%) were added to afford a catalytic system (0.5 ml) containing of cells (10 g l^{-1}) and glucose (1%). Then, *n*-dodecane (50 µl) and the stock solution of **Ru3** (5-10 µl) were added to the reaction tube. The stock solutions of **4a-4c** (2-4 µl, 250 mM in EtOH) were added last. The reaction tubes were sealed and incubated at 250 rpm, 30 °C for 24 h. For the cascades in the sequential mode, **Ru3** (5-10 µl) was added at 12 h. Upon completion, the reaction tubes were incubated on ice for 15-20 min prior to opening these (to minimize the loss of volatile alkenes) and EtOAc (1 ml, containing 1 mM of acetophenone as internal standard) was added. The **1a-1c** and **2a-2c** were extracted, dried over Na_2SO_4 and analysed by GC-MS. To quantify the formation of **4a-4c** and **3a-3c**, the reaction mixtures were acidified with HCl (25 µl, 10 M) and saturated with NaCl. EtOAc (1 ml, containing 1 mM of acetophenone as internal standard) was added to extract **4a-4c** and **3a-3c**. The EtOAc containing **4a-4c** and **3a-3c** was dried over Na_2SO_4, derivatized and analysed by GC-MS.

Procedure for Chemoenzymatic Conversion of 6 to 1a-1c

Freshly harvested *E. coli* (UndB), *E. coli* (C9), and *E. coli* (C10) cells were resuspended in KP buffer (200 mM, pH 8.0) to prepare the stock solutions of cells. The density of cells (OD_{600}) was determined with a UV-spectrometer. To an air-tight reaction tube (25 ml) with screw-cap, stock solutions of cells, KP buffer (200 mM, pH 8.0) and stock solution of glucose (50%) were added to form a system (0.5 ml) with cells (10 g l^{-1}) and glucose (1%). Then, *n*-dodecane (50 µl) and the stock solution of **Ru3** (5-10 µl) were added to the reaction tube. The stock solution of **6** (2-4 µl, 250 mM in EtOH) was added last. The reaction tubes were sealed and incubated at 250 rpm, 30 °C for 24 h. For the cascades in the sequential mode, about NaOH (2 µl, 10 M) was added at 12 h to maintain the pH of the reaction system at 8. Then, *E. coli* (UndB) cells, *n*-dodecane (50 µl), **Ru3** (5-10 µl), and glucose (10 µl, 50%) were added at 12 h. Upon completion, the reaction tubes were incubated on ice for 15-20 min prior to opening these (to minimize the loss of volatile

alkenes) and EtOAc (1 ml, containing 1 mM of acetophenone as internal standard) was added. The **1a-1c** and **2a-2c** were extracted, dried over Na_2SO_4 and analysed by GC-MS.

Procedure for Chemoenzymatic Conversion of 7 to 1a-1c

Lyophilized *E. coli* (TLL) cells were resuspended in KP buffer (200 mM, pH 8.0) to prepare the stock solution of cells (1 g l^{-1}). The cell solution (400 µl) and **7** (5 µl, 100 g l^{-1} emulsion in EtOH) were added to an air-tight reaction tube (25 ml) with screw cap. The reactions were incubated at 250 rpm, 30 °C for 1 h. Freshly harvested *E. coli* (C9) and *E. coli* (C10) cells were resuspended in KP buffer (200 mM, pH 8.0, 1% glucose) to a cell density of 50 g l^{-1}. The stock solution of *E. coli* (C9) and *E. coli* (C10) (100 µl) was added to the reaction mixture at 1 h, and the reaction continued at 250 rpm, 30 °C for 12 h. At 12 h, about NaOH (2 µl, 10 M) was added to maintain the pH of the reaction system at 8. Then, freshly harvested *E. coli* (UndB) cells, *n*-dodecane (50 µl), **Ru3** (5-10 µl), and glucose (10 µl, 50%) were added at 12 h, and reaction continued at 250 rpm, 30 °C for 24 h. Upon completion, the reaction tubes were incubated on ice for 15-20 min prior to opening these (to minimize the loss of volatile alkenes) and EtOAc (1 ml, containing 1 mM of acetophenone as internal standard) was added. The compounds **1a-1c** and **2a-2c** were extracted, dried over Na_2SO_4 and analysed by GC-MS.

Supplementary References

1. Song, J. W. et al. Multistep enzymatic synthesis of long-chain α, ω-dicarboxylic and ω-hydroxycarboxylic acids from renewable fatty acids and plant oils. *Angew. Chem. Int. Ed.* **52**, 2534–2537 (2013).
2. Cha, H. J. et al. Simultaneous enzyme/whole-cell biotransformation of C18 ricinoleic acid into (R)-3-hydroxynonanoic acid, 9-hydroxynonanoic acid, and 1, 9-nonanedioic acid. *Adv. Synth. Catal.* **360**, 696–703 (2018).
3. Dennig, A. et al. Oxidative decarboxylation of short-chain fatty acids to 1-alkenes. *Angew. Chem. Int. Ed.* **54**, 8819–8822 (2015).
4. Rui, Z. et al. Microbial biosynthesis of medium-chain 1-alkenes by a nonheme iron oxidase. *Proc. Natl. Acad. Sci. U. S. A.* **111**, 18237–18242 (2014).
5. Rui, Z., Harris, N. C., Zhu, X., Huang, W. & Zhang, W. Discovery of a family of desaturase-like enzymes for 1-alkene biosynthesis. *ACS Catal.* **5**, 7091–7094 (2015).

6. Kirschner, A., Altenbuchner, J. & Bornscheuer, U. T. Cloning, expression, and characterization of a Baeyer–Villiger monooxygenase from Pseudomonas fluorescens DSM 50106 in E. coli. *Appl. Microbiol. Biotechnol.* **73**, 1065–1072 (2007).
7. Rehdorf, J., Kirschner, A. & Bornscheuer, U. T. Cloning, expression and characterization of a Baeyer-Villiger monooxygenase from Pseudomonas putida KT2440. *Biotechnol. Lett.* **29**, 1393–1398 (2007).
8. Jeon, E. Y. et al. Simultaneous enzyme/whole-cell biotransformation of plant oils into C9 carboxylic acids. *ACS Catal.* **6**, 7547–7553 (2016).
9. Fernandez-Lafuente, R. Lipase from Thermomyces lanuginosus: uses and prospects as an industrial biocatalyst. *J. Mol. Catal. B Enzym.* **62**, 197–212 (2010).
10. Cheng, Q., Thomas, S. M., Kostichka, K., Valentine, J. R. & Nagarajan, V. Genetic analysis of a gene cluster for cyclohexanol oxidation in Acinetobacter sp. strain SE19 by in vitro transposition. *J. Bacteriol.* **182**, 4744–4751 (2000).
11. Salis, H. M., Mirsky, E. A. & Voigt, C. A. Automated design of synthetic ribosome binding sites to control protein expression. *Nat. Biotechnol.* **27**, 946–950 (2009).
12. Espah Borujeni, A., Channarasappa, A. S. & Salis, H. M. Translation rate is controlled by coupled trade-offs between site accessibility, selective RNA unfolding and sliding at upstream standby sites. *Nucleic Acids Res*. **42**, 2646–2659 (2013).
13. Farasat, I. et al. Efficient search, mapping, and optimization of multi‐protein genetic systems in diverse bacteria. *Mol. Syst. Biol.* **10**, 731 (2014).
14. Jeschek, M., Gerngross, D. & Panke, S. Rationally reduced libraries for combinatorial pathway optimization minimizing experimental effort. *Nat. Commun.* **7**, 11163 (2016).
